# Spatiotemporal Tracking of Three Novel Transposable Element Invasions in *Drosophila melanogaster* over the Last 30 Years

**DOI:** 10.1093/molbev/msaf143

**Published:** 2025-06-06

**Authors:** Riccardo Pianezza, Almorò Scarpa, Anna Haider, Sarah Signor, Robert Kofler

**Affiliations:** Institut für Populationsgenetik, Vetmeduni Vienna, Veterinärplatz 1, Vienna 1210, Austria; Vienna Graduate School of Population Genetics, Vetmeduni Vienna, Vienna, Austria; Institut für Populationsgenetik, Vetmeduni Vienna, Veterinärplatz 1, Vienna 1210, Austria; Vienna Graduate School of Population Genetics, Vetmeduni Vienna, Vienna, Austria; Institut für Populationsgenetik, Vetmeduni Vienna, Veterinärplatz 1, Vienna 1210, Austria; Biological Sciences, North Dakota State University, Fargo, ND, USA; Institut für Populationsgenetik, Vetmeduni Vienna, Veterinärplatz 1, Vienna 1210, Austria

**Keywords:** transposable elements, Drosophila, transposon invasions, horizontal gene transfer, genome evolution

## Abstract

Transposable elements (TEs) are repetitive sequences capable of mobilizing within genomes, exerting a significant influence on evolution throughout the tree of life. Using a novel approach that does not require prior knowledge of the sequence of repeats, we identified three novel TE invasions in *Drosophila melanogaster*: *McLE* spread between 1990–2000, *Souslik* between 2009–2012, and *Transib1* between 2013–2016. We recapitulate previous findings, revealing that a total of 11 TEs invaded *D. melanogaster* over the past two centuries. These 11 invasions increased the fly genome by ∼1 Mbp. Using data from over 1,400 arthropod genomes, we provide evidence that these TE invasions were triggered by horizontal transfers, with *Drosophila simulans* and species of the *Drosophila willistoni* group acting as putative donors. Through the analysis of ∼600 short-read datasets spanning diverse geographic regions, we reveal the rapidity of TE invasions: *Transib1* swiftly multiplied from three isolated epicenters in 2014 to all investigated populations in just 2 years. Our findings suggest that anthropogenic activities, which facilitate the range and population expansions of *D. melanogaster*, could have accelerated the rate of horizontal transposon transfer as well as the spread of the TEs into the worldwide population. Given the significant impact of TEs on evolution and the potential involvement of humans in their dispersal, our research has crucial implications for both evolution and ecology.

Significance Transposable elements (TEs) are DNA sequences that selfishly propagate within a species. To persist, TEs are occasionally transmitted to naive species by horizontal transfer (HT), a process once thought to be extremely rare. During the past two decades, studies have increasingly shown that HT is more common than previously thought. Our work builds on this growing body of evidence by showing that three TEs invaded the genomes of *Drosophila melanogaster* in the past 30 years. Notably, we show that one of these TEs, *Transib1*, spread globally within just 2 years. Together with previously reported cases, 11 TEs invaded *D. melanogaster* during the last 200 years. These 11 TEs increase the fly genome by approximately 1%, underscoring the role of selfish DNA invasions as a major force driving the evolution of fly genomes.

## Introduction

Human activity is reshaping the global distribution of species at unprecedented rates, with climate change emerging as a significant consequence of anthropogenic influence (Turbelin et al. 2017; Gutierrez and Ponti 2022). As species move into new environments, so do their pathogens, changing the assemblage of organisms that are now being exposed to these pathogens (Bebber et al. 2013). Transposable elements (TEs), i.e. short stretches of DNA capable of multiplying within genomes, may also be affected by these dynamics. As the species distributions change, previously isolated species come into contact, creating new opportunities for TEs to spread by horizontal transfer (HT; i.e. the transfer of heritable genetic material between species) (Engels 1992; Pianezza et al. 2024; Scarpa et al. 2025). TEs are generally categorized based on the transposition mechanism into two main classes: TEs that replicate through a “copy-and-paste” process involving an RNA intermediate (retrotransposons) and TEs that move via a “cut-and-paste” mechanism (DNA transposons) (Finnegan 1989; Wicker et al. 2007). While some insertions are likely beneficial, it is thought that most TE insertions are either neutral or deleterious to the host (Aminetzach et al. 2005; Mateo et al. 2014; Arkhipova 2018). To suppress the movement of TEs, hosts have developed dedicated defense mechanisms frequently involving small RNAs (Sarkies et al. 2015), although defence mechanisms exist that do not require small RNAs (Gladyshev 2017; Seczynska and Lehner 2023). For example, in *Drosophila*, small RNAs, termed piRNAs, suppress TEs both transcriptionally and posttranscriptionally (Brennecke et al. 2007; Le Thomas et al. 2013). TEs that are silenced by the host will then gradually accumulate mutations, ultimately leading to their immobilization and potential loss over time. One strategy to escape this gradual decay is the HT to a species lacking the TE (Schaack et al. 2010; Peccoud et al. 2017).

HT of TEs among insects has been observed to be widespread, with most of the evidence being indirect, such as a higher than expected sequence identity of TEs between two species or a patchy phylogenetic distribution of the TEs (Loreto et al. 2008 ; Bartolomé et al. 2009; Wallau et al. 2012 , 2016, 2018; Peccoud et al. 2018). Direct observations of TE invasions have rarely been documented. The first well-documented case is the invasion of the *P*-element in *Drosophila melanogaster* between 1950–1980, following HT from *Drosophila willistoni* (Daniels et al. 1990). This event was only possible because *D. melanogaster* extended its range into the Americas about 200 years ago, establishing contact with *D. willistoni*, a species that is endemic to South and Central America (Engels 1983; Keller 2007). Later, it was shown that the *I*-element, *Hobo* and *Tirant* also spread in *D. melanogaster* populations between 1930 and 1960 (Bucheton et al. 1992; Bonnivard et al. 2000; Schwarz et al. 2021). Analyzing the genomes of historical *D. melanogaster* specimens from museum collections, we showed that three additional TEs—*Blood*, *Opus*, and *412*—spread in *D. melanogaster* between 1850 and 1930 (Scarpa et al. 2023). This is in agreement with previous studies suggesting that these TEs were horizontally exchanged between *D. melanogaster* and *Drosophila simulans* (Sánchez-Gracia et al. 2005; Bartolomé et al. 2009; Modolo et al. 2014). Based on a repeat library generated for different *Drosophila* species (Chakraborty et al. 2021), we recently showed that *Spoink* invaded *D. melanogaster* between 1983 and 1993 (Pianezza et al. 2024), increasing the number of TEs that spread in *D. melanogaster* in the last 200 years to eight. Here, we ask if additional TEs not found in any of the extant repeat libraries could have invaded *D. melanogaster* populations. To identify all recent TE invasions, we need to employ an approach that does not rely on any repeat library. We reasoned that TE invasions should lead to a recognizable pattern, such that sequences present in the genomes of recently collected strains should be absent in older ones. Using this approach, we have indeed found that three TEs invaded natural *D. melanogaster* populations during the last 30 years.

We show that *McLE* (*Micropia*-like-element), a LTR retrotransposon of the *gypsy*/*mdg3* superfamily, spread in *D. melanogaster* between 1990 and 2000, as well as *Souslik*, a LTR retrotransposon likely of the *gyspy*/*osvaldo* superfamily, between 2009 and 2012 and *Transib1*, a DNA transposon, between 2013 and 2016. We show that the *McLE* invasion was triggered by a HT from a species of the *willistoni* group, whereas the *Transib1* and *Souslik* invasions were triggered by a HT from the closely related *D. simulans*. Based on 585 samples collected during the invasions from different geographic regions, we were able to trace the spatiotemporal dispersal of *Transib1*. The invasion of *Transib1* began in at least three geographically isolated epicenters around 2014 (France, Ukraine, North America). Within a mere 2 years, *Transib1* spread to all investigated populations, demonstrating that TEs can rapidly infect geographically distinct populations.

## Results

### *McLE*, *Souslik*, and *Transib1* Recently Spread in the *D. melanogaster* Genome

**Fig. 1.**
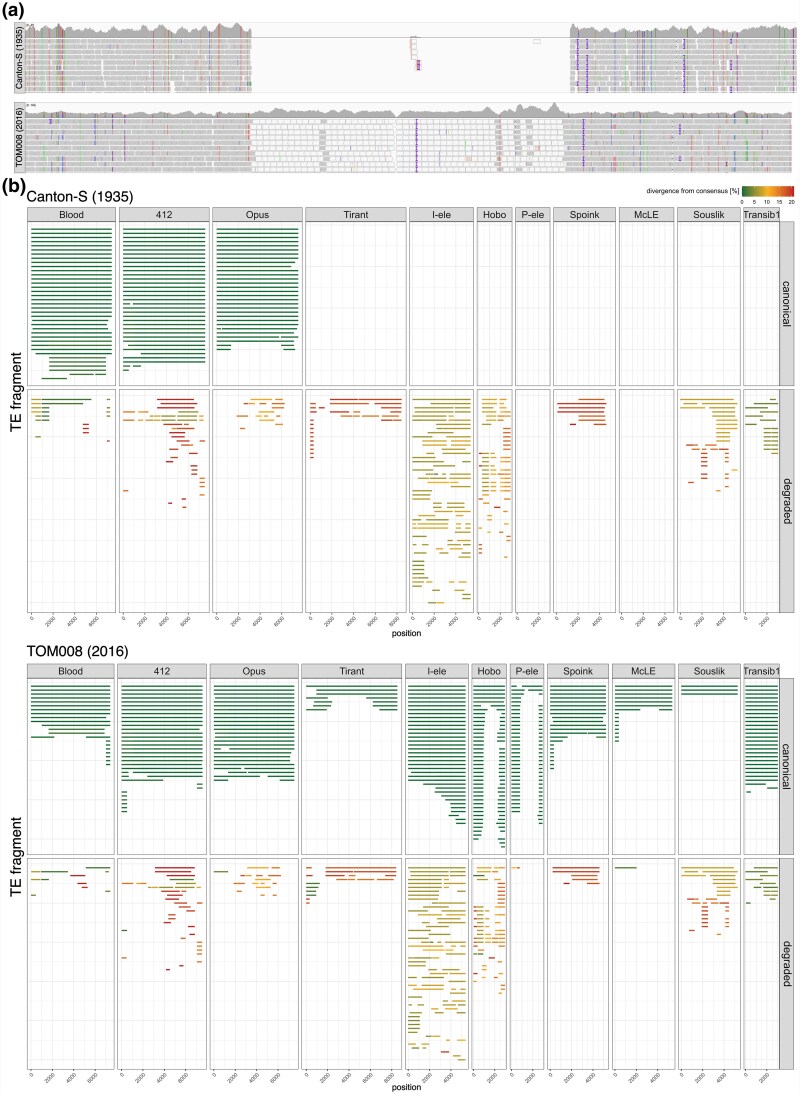
Overview of the HTs of TEs in *D. melanogaster* during the last two centuries from long-read assemblies. a) Illustration of our approach to find TE invasions. A novel TE invasion can be detected by gaps in the coverage, when reads of an old strain (e.g. *Canton-S*, sampled 1935) are aligned to the assembly of a recent strain (e.g. Tom008; sampled 2016; top panel). The gap in the example is due to *Souslik*. By contrast, no coverage gap is found if reads from the same strain are aligned (bottom panel). IGV was used for the visualization (Robinson et al. 2011). b) Overview of sequences with similarity to the 11 TEs in the long-read assemblies of Canton-S and Tom008. Horizontal bars represent the matching regions between the TE insertions and the consensus sequence. Colors represent divergence from the consensus sequence. Based on a divergence threshold of 1.5%, we refer to the fragments as canonical (≤1.5%) and degraded (>1.5%). Note that canonical insertions are present in the young but absent in the old strain. The first three TEs spread before 1935, which explains the presence of canonical insertions in both strains.

Using a repeat library for species of the *Drosophila simulans* complex (Chakraborty et al. 2021), we previously discovered that the TE *Spoink* invaded *D. melanogaster* between 1983 and 1993 (Pianezza et al. 2024). This raised the question of whether other TEs, absent or incompletely represented in current repeat libraries, may have spread more recently in *D. melanogaster*. A major limitation in the identification of novel invasions is the need for a library of known TEs. We sought to circumvent this by developing an approach which can identify new TE insertions without a reference library. We reasoned that short reads from an old strain (before an invasion), aligned to an assembly of a recently collected strain (after an invasion), should result in coverage gaps at insertion sites of novel TEs (Fig. 1a; supplementary figs. S1 and S2, Supplementary Material online). To detect signatures of novel invasions, we developed a software called GenomeDelta, which aligns short reads, identifies coverage gaps (corresponding to the individual insertions of a novel TE family), extracts their sequences, and clusters them by sequence identity (Pianezza et al. 2024).

Here, we aligned reads of *Canton-S*, which was sampled in 1935, to the assembly of *TOM008*, a strain collected in 2016 (Lindsley and Grell 1968; Rech et al. 2022). We identified several sequences which were causing coverage gaps, many of which serve as a proof-of-concept as they corresponded to known invasions that occurred after 1935: *Tirant*, the *I*-element, *Hobo*, the *P*-element, and *Spoink* (some examples are shown in supplementary fig. S1, Supplementary Material online). Interestingly, we discovered three additional TE sequences leading to coverage gaps (Fig. 1a; supplementary fig. S1, Supplementary Material online). Comparing two additional short-read datasets of strains collected before 1965 to three additional long-read assemblies of strains collected in 2015–2016 with GenomeDelta did not lead to the detection of additional TEs.

The first sequence corresponds to *McLE*, which was originally identified in two assemblies from the *Drosophila* Genetic Reference Panel, which were sampled in 2003 in Raleigh (GenBank MN418888; Mackay et al. 2012; Ellison and Cao 2020). As insertions of *McLE* were absent in the reference strain *Iso-1*, the authors suggested that *McLE* recently invaded the *Drosophila* Genetic Reference population. *McLE* is a 5,360 bp LTR retrotransposon with an LTR of 361 bp. It has roughly 70% sequence identity with the consensus sequence of *Micropia* annotated in *D. melanogaster* (Quesneville et al. 2005). Based on the alignment of the reverse transcriptase domain, *McLE* is a member of the *gypsy*/*mdg3* group, similarly to *Spoink* (supplementary fig. S3, Supplementary Material online; Pianezza et al. 2024). *McLE* and *Spoink* have a different length (5,360 vs. 5,216 bp), different sized LTRs (361 vs. 349 bp), and generally share little sequence identity (63% identical). In addition, *McLE* and *Spoink* insertions are at different genomic sites (supplementary fig. S4, Supplementary Material online).

The second sequence identified by GenomeDelta shares 99.68% sequence identity with *Souslik*, a TE previously described in *D. simulans* (Glukhov et al. 2016). *Souslik* has a length of 5,275 bp and an LTR of 210 bp. *Souslik* is a member of the *Ty3*/*Gypsy* group and encodes a *gag*-*pol* fusion product, but no *env*. The classification of *Souslik* as *Ty3*/*Gypsy* element is also in agreement with a previous work (Bargues and Lerat 2017) (termed “*Sacco*,” which has 97% sequence identity with *Souslik*). The phylogenetic tree based on the reverse transcriptase domain suggests that *Souslik* is a member of the *gyspy*/*osvaldo* group (supplementary fig. S3, Supplementary Material online). However, *Souslik* classification remains controversial, with some studies linking it to *Mag* elements (Glukhov et al. 2016), while others place it in the *micropia/sacco* group based on *pol* sequence (Bargues and Lerat 2017).

Finally, the third sequence has a high identity with *Transib1* in *D. melanogaster* (96.06% over 72% of the length) and *D. simulans* (99.93% over 100% of the length). *Transib1* is a DNA transposon of length 3,030 bp. The *Transib1* sequence in *D. melanogaster* was reconstructed from transposon fragments scattered throughout the genome. Before this study, no full-length *Transib1* insertion had been identified in *D. melanogaster* (Kapitonov and Jurka 2003).

To further investigate these recent invasions, we analyzed TE insertions in long-read assemblies of *D. melanogaster* strains collected at different times during the last century (*Canton-S* 1935 and *Tom008* 2016). Using RepeatMasker, we identified sequences with similarity to any of the 11 TEs that may have invaded *D. melanogaster* recently [Fig. 1b; *Blood*, *Opus*, *412*, *Tirant*, *I*-element, *Hobo*, *P*-element, *Spoink*, *McLE*, *Souslik*, *Transib1*; for alternative names of these TEs in Dfam (Hubley et al. 2016) and the repeat library of Rech et al. (2022) see supplementary table S1, Supplementary Material online]. We refer to insertions with high identity to the consensus sequence (≤1.5%) as canonical insertions, to differentiate them from degraded copies (>1.5%). The choice of the threshold (1.5%) is arbitrary but useful, as it allows distinguishing old insertions (degraded; present before 1935) from recent ones (canonical; present after 1935; Fig. 1b). As expected, in *TOM008* (2016), we found canonical insertions for all eight TEs that were previously shown to have spread in *D. melanogaster* in the last two centuries. Canonical insertions were absent in *Canton-S* (1935) for the five TEs that spread after 1935, i.e. *Tirant*, *I*-element, *Hobo*, *P*-element, and *Spoink*. Since *Blood*, *Opus*, and *412* spread between 1850 and 1933 (Scarpa et al. 2023), canonical insertions of these TEs were present in both strains (*Canton-S* and *TOM008*). No high-quality long-read based assembly is available for any strain sampled before 1925–1935 (i.e. before *Canton-S* and *Oregon-R* were sampled). Importantly, we found canonical insertions of *McLE*, *Souslik* and *Transib1* in *TOM008* (2016) but not in *Canton-S* (1935; Fig. 1b). Degraded fragments were found for all TEs, except for the *P*-element and *McLE*, in both *TOM008* and *Canton-S* (Fig. 1b). Ancient invasions of TEs sharing some sequence identity with the focal TE are the most probable source of these degraded fragments. These ancient invasions were likely triggered by independent HT events. For example, the degraded *Transib1* fragments present in strains collected before 2015 (such as Iso-1) are likely due to a more ancient HT (Modolo et al. 2014).

An investigation of the insertion sites reveals that the degraded fragments of the TEs are close to centromeric heterochromatin, whereas the canonical insertions are distributed over the entire chromosome arm (supplementary fig. S4, Supplementary Material online). An analysis of the assemblies of multiple strains collected between 1968 and 2015 confirms that the canonical insertions of *McLE*, *Souslik*, and *Transib1* gradually emerged during the last decades, while degraded fragments are present in all analysed strains (supplementary fig. S5, Supplementary Material online). Next, we analyzed the copy number of all TEs in several sequenced strains sampled around 1980 and 2015 with DeviaTE (Weilguny and Kofler 2019). For each TE family, DeviaTE normalizes the average coverage of a TE to the average coverage of single copy genes, which allows inferring the TE copy number per haploid genome. An analysis of the read coverage shows that *McLE*, *Souslik*, and *Transib1* have a uniformly elevated coverage in strains sampled in 2016, compared to strains sampled before 1968 (supplementary figs. S6, S7, and S8, Supplementary Material online). For *Souslik* and *Transib1*, a low level of coverage based on diverged reads can also be found in strains sampled before 1968, in agreement with the presence of degraded fragments in all analysed strains (supplementary figs. S6, S7, and S8, Supplementary Material online).

Finally, investigating the population frequency of the TE insertions, we found that canonical insertions are largely segregating at low frequency, whereas degraded insertions are mostly fixed (supplementary fig. S9, Supplementary Material online). This is consistent with the hypothesis that canonical insertions are recent, whereas degraded insertions are more ancient.

In summary, we suggest that the LTR-retrotransposons *McLE* and *Souslik*, as well as the DNA transposon *Transib1*, recently spread in the *D. melanogaster* genome. Degraded fragments, likely remnants of past invasions, can be found in all analysed strains for *Souslik* and *Transib1*, but not for *McLE*.

### History of TE Invasions in *D. melanogaster* During the Past Two Centuries

Given that our unbiased approach (GenomeDelta) likely detected all recent TE invasions, we can now reconstruct the complete history of TE invasions in *D. melanogaster* over the last two centuries. We analyzed 585 short-read datasets from individual strains or pooled populations collected during the last two centuries (supplementary file S1, Supplementary Material online). We inferred the invasion status from two key metrics, i.e. the TE copy numbers and the frequency of SNPs diagnostic for canonical insertions. For each of the 11 TEs that recently spread in *D. melanogaster*, we identified a set of diagnostic SNPs that are present in canonical insertions but absent in degraded ones (see supplementary fig. S10, Supplementary Material online). The frequency of these diagnostic SNPs provides an approximate measure of the proportion of canonical insertions within the TE family. For instance, a frequency of 0.8 indicates that approximately 80% of the insertions are canonical, while the remaining 20% are degraded. To estimate the abundance of canonical insertions for each TE, we averaged the frequencies of all diagnostic SNPs for that TE (see supplementary fig. S11, Supplementary Material online; for a list of all diagnostic SNPs see supplementary file S5, Supplementary Material online). For TEs where we did not find diagnostic SNPs (no short reads aligning to the TE in old samples, i.e. the *P*-element and *Opus*), we used frequency estimates of 1.0 (presence of reads) and 0.0 (absence of reads).

**Fig. 2.**
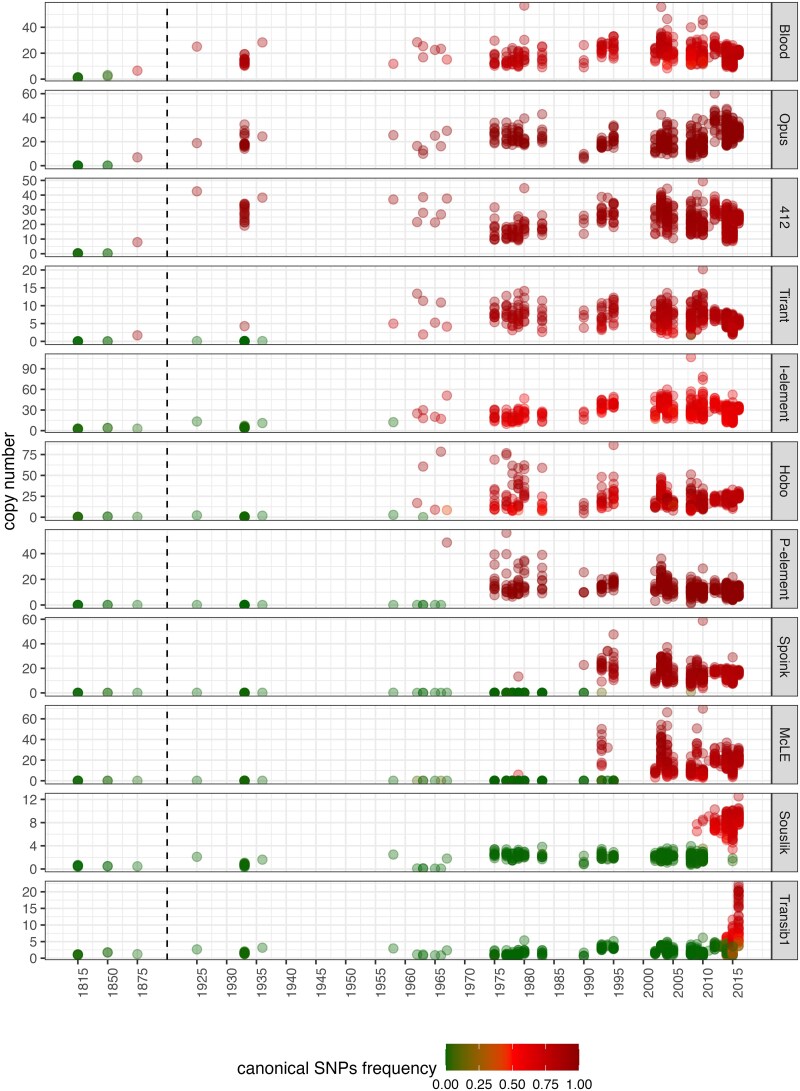
Complete invasion history of *D. melanogaster* during the last two centuries. We tracked the copy numbers of TEs (*y*-axis) and the frequency of SNPs diagnostic for canonical insertions (color) in *D. melanogaster* strains and pooled populations sampled over the last two centuries. A recent invasion is indicated by a sharp increase in two key metrics, i.e. the copy numbers of the TE and the frequency of canonical SNPs (which captures the ratio of canonical to degraded insertions for a given TE family).

Consistent with previous works, both of our key metrics (copy number and diagnostic SNPs) suggest that *Blood*, *Opus* and *412* spread between 1850 and 1933, followed by *Tirant* (1933–1950), the *I*-element (1933–1950), *Hobo* (∼1950), the *P*-element (∼1960), and *Spoink* which spread between 1983–1993 (Fig. 2; Daniels et al. 1990; Bucheton et al. 1992; Bonnivard et al. 2000; Schwarz et al. 2021; Scarpa et al. 2023; Pianezza et al. 2024). Our data further suggest that *McLE* spread between 1990 and 2000, slightly after *Spoink*. *Souslik* spread between 2009–2012, and *Transib1* between 2013–2016 (Fig. 2).

Next, we traced the invasion of these 11 TEs in 49 long-read assemblies of *D. melanogaster* strains collected between 1925 and 2018 (supplementary file S2, Supplementary Material online). A major advantage of long-read assemblies is that the copy number of canonical insertions can be directly inferred from the RepeatMasker output. However, a drawback is the lower temporal resolution compared to short-read data, due to the limited number of high-quality assemblies. The abundance of canonical insertions in long-read assemblies reproduces the timing of the TEs previously shown to have spread in *D. melanogaster* between 1925 and 2018, i.e.: *Tirant*, *I*-element, *Hobo*, *P*-element and *Spoink* (supplementary fig. S12, Supplementary Material online). Interestingly, canonical *Tirant* insertions are still absent in a few recently collected strains (supplementary fig. S12, Supplementary Material online). No canonical insertions of *McLE*, *Souslik* and *Transib1* were found in any strain collected before 2002 (supplementary fig. S12, Supplementary Material online). The first canonical *McLE* insertions are found in strains collected in 2003, for *Souslik* in 2011 and for *Transib1* in 2015. The abundance of canonical insertions in long-read assemblies is thus consistent with the timing of the invasion inferred from the short-read data.

In summary, we inferred the complete invasion history for all 11 TEs that spread in *D. melanogaster* between 1810 and 2018. We were able to reproduce the timing of the eight TEs previously shown to have spread in *D. melanogaster* recently, and showed that *McLE* spread between 1990 and 2000, *Souslik* between 2009 and 2012, and *Transib1* between 2013 and 2016

### Geographic Spread of the Invasions

**Fig. 3.**
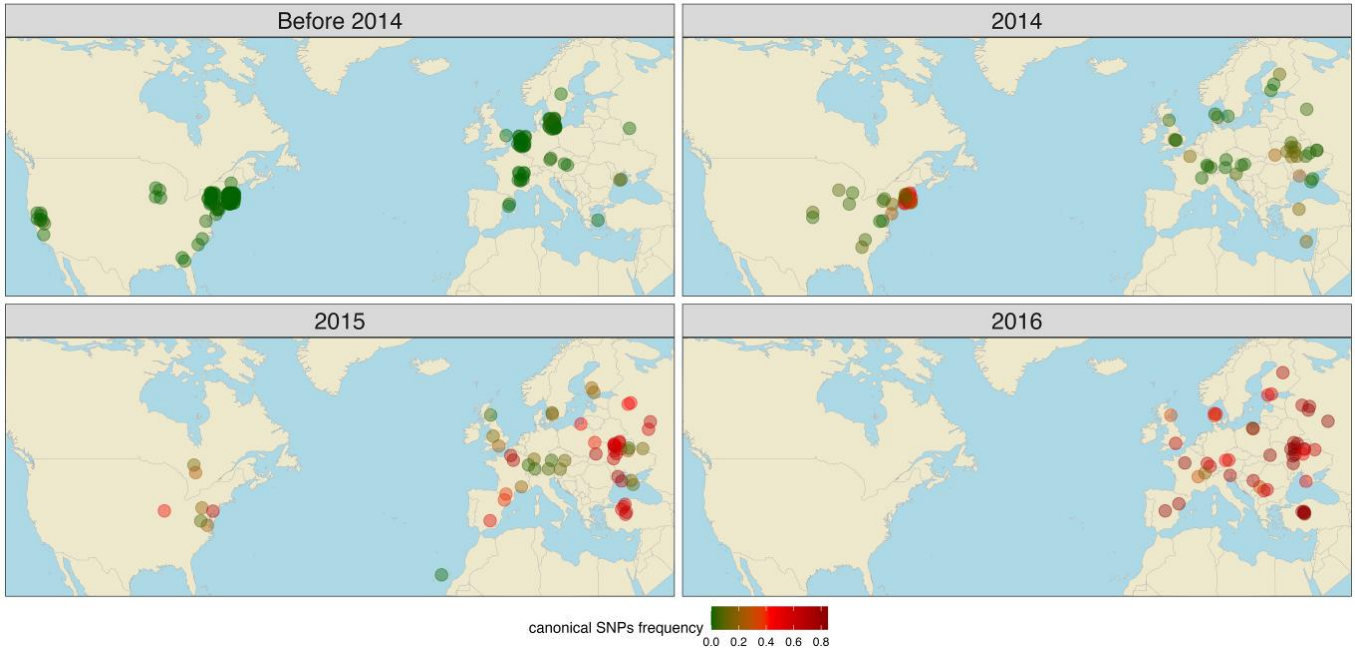
Geographic spread of canonical *Transib1* insertions in *D. melanogaster* samples (strains or pooled populations) collected from Europe and North America between 2013 and 2016. Colors indicate the frequency of SNPs diagnostic for the presence of the canonical *Transib1* insertions.

Next, we examined the timing of the geographical spread of the TEs across worldwide populations. Given the recent spread of *McLE*, *Souslik*, and *Transib1* over the last few decades, there is a wealth of resources available to infer the timing of these invasions. In particular, consortia such as DrosEU (mostly Europe), DrosRTEC (mostly North America), and DGN (worldwide) contributed a vast number of short-read datasets of strains or pooled populations sampled from different locations during the last decades (Lack et al. 2015, 2016; Kapun et al. 2021; Machado et al. 2021). Using these rich resources, we investigated the spatiotemporal spread of *McLE*, *Souslik*, and *Transib1*, relying on the diagnostic SNPs (Fig. 3; supplementary fig. S13, Supplementary Material online).

The first population in which we detected *McLE* was an African population collected in 1993, when it was not yet present in a population from China. After 1996, *McLE* was found in populations across the globe (supplementary fig. S13, Supplementary Material online). Note that the early presence of *McLE* in Africa does not imply that the invasion started in Africa, as too few data from other populations are available between 1993–1995 to reliably infer the origin of the invasion (other lines of evidence suggest that the invasion started in South America; see below). Based on the abundance of canonical insertions in long-read assemblies, we confirm that *McLE* was absent in worldwide *D. melanogaster* strains before 1995 but present afterwards (supplementary fig. S14, Supplementary Material online).

Global populations lacked *Souslik* insertions before 2009. *Souslik* was first found in North America in 2009 and had become widespread in most populations by 2011. However, in 2015, *Souslik* was still absent in populations sampled from the Caribbean archipelago of Guadeloupe (GS_Sai_15_2 and GS_Des_15_1; supplementary fig. S13, Supplementary Material online). This shows that geographic isolation of island populations may protect them, at least for some time, from TE invasions. The long-read assemblies confirm that canonical *Souslik* insertions were absent in global *D. melanogaster* strains sampled before 2003 and present afterwards (supplementary fig. S14, Supplementary Material online).

The timing of the *Transib1* invasion was particularly fortunate, as it fell right into the period when DrosEU and DrosRTEC were collecting massive amounts of samples from Europe and North America (2013–2016; Fig. 3). This allowed us to trace the *Transib1* invasion with unprecedented spatiotemporal resolution (Fig. 3). *Transib1* was totally absent in populations from both North America and Europe before 2014. In 2014, a few *Transib1* copies were found in populations from Virginia, France, and Ukraine. However, the vast majority of the samples did not have *Transib1* insertions by 2014. In 2015, the *Transib1* invasion progressed rapidly, with *Transib1* insertions found in many populations from both North America and Europe. By 2016, all sampled populations had *Transib1* insertions (Fig. 3). The spread of *Transib1* was extremely rapid. Within just 2 years (2014–2016), it spread from a few populations in Europe and North America to all sampled populations. It is notable that the invasion did not spread from a single location but started at multiple geographically isolated “epicenters” (i.e. Ukraine, France, Virginia). Human activity (e.g. air traffic) may be responsible for this rapid spread of *Transib1* among isolated populations. The long-read assemblies confirm that canonical *Transib1* insertions are absent in all strains sampled before 2011 and present in strains sampled later (supplementary fig. S14, Supplementary Material online).

In summary, we were able to trace the spatiotemporal spread of *McLE*, *Souslik*, and *Transib1*. The invasion of *Transib1* is particularly important, as it demonstrated that the spread of this TE occurred concurrently from several geographic locations, and that within just 2 years the majority of the populations were infected. This extremely rapid dispersal of a TE has not been previously documented, as sampling of flies from the wild is generally not dense enough.

### Origin of *McLE*, *Souslik*, and *Transib1*

**Fig. 4.**
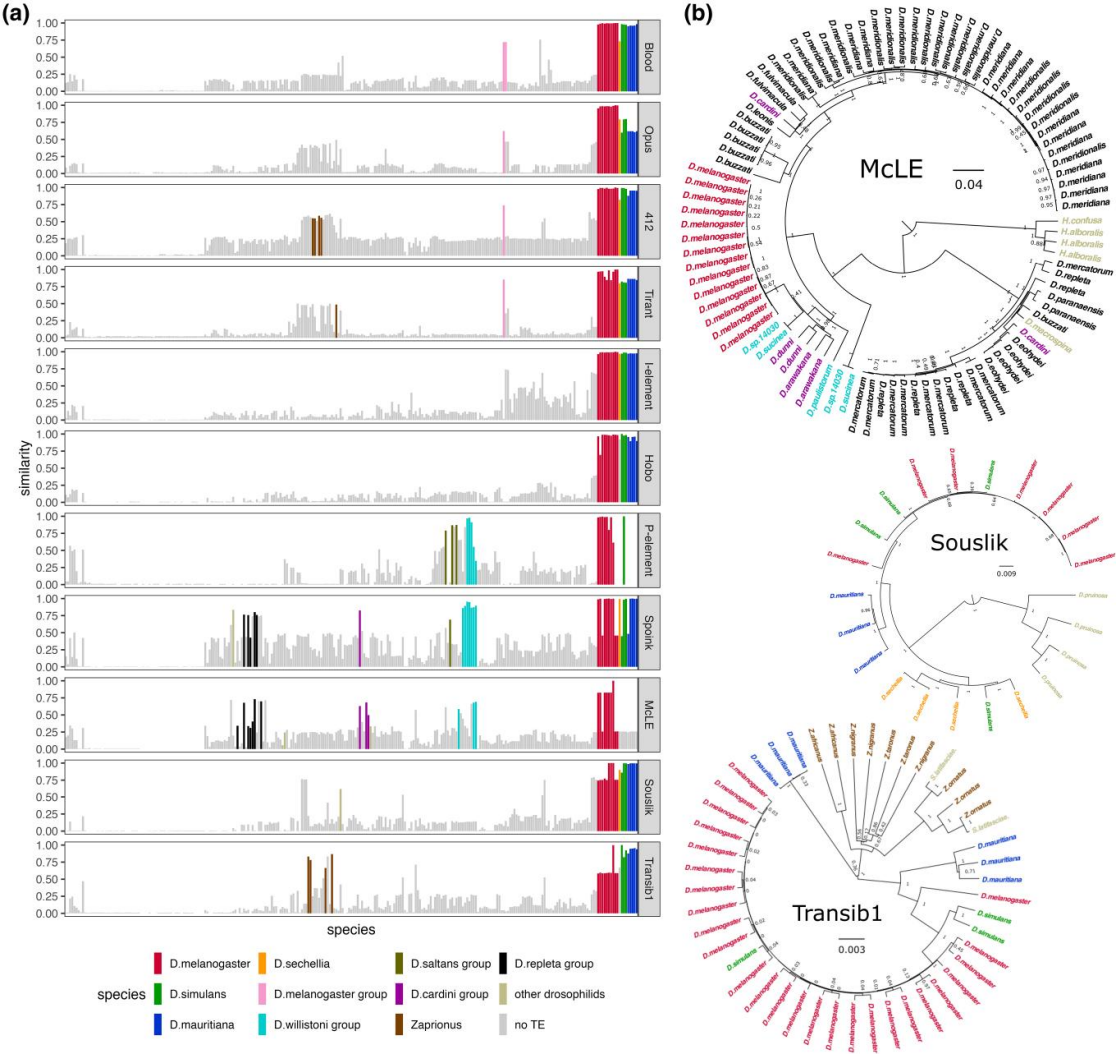
The *Souslik* and *Transib1* invasions were triggered by HT from *D. simulans*, whereas *McLE* was transferred from a species of the *willistoni* group. a) Similarity of the 11 TEs that recently invaded *D. melanogaster* with insertions in 266 high-quality assemblies of 242 *Drosophila* species. The barplots show, for each species, the similarity between the given TE and the best match in an assembly. The species were arranged by relatedness. Colors indicate species or species groups with full-length insertions (>80% of length). b) Bayesian tree of *McLE*, *Souslik*, and *Transib1* insertions in species having at least one full-length insertion.

The invasions of *McLE*, *Souslik*, and *Transib1* in *D. melanogaster* were probably triggered by an HT event from a different species. To identify the origin of the HT, we investigated TE insertions in the assemblies of 266 drosophilids (Kim et al. 2021, 2023) (supplementary file S3, Supplementary Material online), representing 242 species. Additionally, we analyzed the reference genomes of 1,226 arthropods species (supplementary file S4, Supplementary Material online). We used RepeatMasker to identify *McLE*, *Souslik*, and *Transib1* insertions in these genomes, together with the other 8 previously reported invaders (Fig. 4a). In agreement with previous work, we found insertions with a high similarity to *Blood*, *Opus*, *412*, *Tirant*, the *I*-element, and *Hobo* in *D. simulans* and related species, whereas the *P*-element and *Spoink* have similar insertions in species of the *willistoni* group (Daniels et al. 1990; Scarpa et al. 2023; Pianezza et al. 2024). For *McLE*, we found highly similar insertions in species of the *willistoni*, *cardini* and *repleta groups*. Interestingly, *McLE* was entirely absent in *D. simulans* and other species of the *simulans* species complex (*D. mauritiana* and *D. sechellia*). For *Souslik* and *Transib1*, the most similar insertions were found in the *simulans* species complex. We did not find sequences with a high similarity to the 11 TEs that recently invaded *D. melanogaster* in the 1,226 arthropods, outside of the drosophilids (supplementary fig. S15, Supplementary Material online; apart from *McLE*, where insertions with some sequence identity can be found in *Anastrepha obliqua* and *Merodon equestris*).

To further elucidate the source of the HT, we generated phylogenetic trees of full-length insertions of *McLE*, *Souslik*, and *Transib1* (Fig. 4b). Insertions of all the three TEs have short branches in *D. melanogaster*, consistent with a recent invasion (Fig. 4b). The *McLE* insertions in *D. melanogaster* are nested in insertions of species of the *willistoni* group. This suggests that, much as the *P*-element and *Spoink*, a species of *willistoni* group acted as donor of *McLE*. Why species of the *willistoni* group repeatedly acted as donors to *D. melanogaster* is an open question. The phylogenetic trees of *Souslik* and *Transib1* suggest a different origin, as their insertions exhibit the closest relationship to *D. simulans*.

To corroborate our results, we statistically tested the hypothesis of a HT of the TEs by comparing the sequence divergence of orthologous genes between *D. melanogaster* and the putative donor species with the divergence of the TEs. For all 11 TEs that spread in *D. melanogaster* during the last 200 years, the sequence divergence is lower than the divergence of 95% of the genes, supporting the HT of the TEs (supplementary fig. S16, Supplementary Material online).

In summary, our findings suggest that *McLE* invaded the genome of *D. melanogaster* through a HT from a species of the *willistoni* group, while *D. simulans* was the donor of *Souslik* and *Transib1*.

## Discussion

Using a novel approach to identify recent genomic invaders, we found that three TEs, *McLE*, *Souslik*, and *Transib1*, spread in *D. melanogaster* populations between 1990 and 2016. All three invasions occurred after the spread of *Spoink* (1983–1993), previously thought to be the most recent invasion (Pianezza et al. 2024). The three invasions were probably triggered by a HT from different species. Most previously reported cases of HT of TEs were inferred from indirect evidence, such as an unusual high sequence identity of the TE among species, a patchy distribution of the TE among closely related species and phylogenetic discrepancy between the TE and the host species (Loreto et al. 2008; Bartolomé et al. 2009; Wallau et al. 2012, 2016, 2018; Peccoud et al. 2018). The three HTs described here are supported by direct and more compelling evidence, i.e. the absence of canonical insertions in old samples and their presence in recently collected samples (e.g. strains or pooled populations). This presence/absence pattern of the canonical insertions is supported by numerous short-read data and long-read assemblies (Fig. 2; supplementary fig. S12, Supplementary Material online).

Combined with the eight previously known cases, our work suggests that in total 11 TEs invaded *D. melanogaster* populations in the last two centuries following HT. Since our method for identifying novel invaders is unbiased, i.e. it does not require prior knowledge about the sequence of repeats, we argue that we identified all TE invasions in *D. melanogaster* populations between ∼1,810 (the earliest collected strain) and 2,018 (the most recently collected long-read assembly). We may have missed only invasions of TEs with a very high sequence identity to resident TEs (which might not result in noticeable coverage gaps). It is not clear if HT of a TE with high identity to resident TEs could actually trigger an invasion, as the piRNA based host defence will likely be active against the resident TE and the piRNAs complementary to the resident TE will also target the novel TE (some mismatches are tolerated Schwarz et al. 2021; Gainetdinov et al. 2023). We may have further missed local invasions, that just happened in isolated populations.

We think it is likely that additional TEs will invade *D. melanogaster* populations in the next decades. It is interesting to speculate which TEs may spread next. Given the high rate of HT events between *D. melanogaster* and *D. simulans*, TEs absent in one of the two species could invade the other. For example, *Shellder* has spread in *D. simulans* and *D. mauritiana*, but it’s still absent in *D. melanogaster* (Scarpa et al. 2025). On the other hand, we found *McLE* to be present in *D. melanogaster* but not in *D. simulans*. Thus, it is a promising candidate for future invasions in *D. simulans* and related species. It is also plausible that additional transposons will jump in *D. melanogaster* from species of the *willistoni* group.

In fact, one of the interesting patterns that has emerged is that the recent invasions in *D. melanogaster* are triggered by HT either from *D. simulans* (*Blood*, *Opus*, *412*, *Tirant*, *I*-element, *Hobo*, *Souslik*, *Transib1*) or a species of the *willistoni* group (*P*-element, *Spoink*, *McLE*). HT of TEs between the closely related *D. simulans* and *D. melanogaster* is not unexpected, as there could be a rare hybridization among some members of the complex. Additionally, the species have a somewhat overlapping ecology (Capy and Gibert 2004) and could share potential vectors (e.g. viruses, parasites and *Wolbachia*) that might mediate the HT of TEs. In agreement with this, previous work found that HT is most likely among closely related insect species (Peccoud et al. 2017). However, *D. melanogaster* and species of the *willistoni* group are distantly related (100 My (Obbard et al. 2012)). Thus, an important open question is why species of the *willistoni* group repeatedly acted as donors of TE invasions in *D. melanogaster*, and not any of the many other species that came into contact with *D. melanogaster* following its range expansion (e.g. *D. pseudoobscura*, which is endemic to North America Dobzhansky and Epling 1944). A related question is whether the horizontal exchange between *D. melanogaster* and species of the *willistoni* group is unidirectional or if species of the *willistoni* group also recently received TEs from *D. melanogaster*. For example, it was suggested that *Copia* was recently horizontally transferred from *D. melanogaster* to *D. willistoni*, although recent works did not confirm this (Jordan et al. 1999; Rubin et al. 2011).

Given the amount of new horizontal transposon transfers in *D. melanogaster* in the last two centuries, another open question concerns the effect of these novel invasions on genome architecture. The 11 invasions increased the genome size of *D. melanogaster* by around 1Mbp during the last two centuries (supplementary table S2, Supplementary Material online). Furthermore, the 11 invasions could also lead to inversions, translocations, or other large genomic rearrangements, as they have in cichlids (Quah et al. 2025). Thus, the 11 new TEs might also have an impact on the organization of the genome of *D. melanogaster*.

An important open question is whether the high rate of recent invasions in the last two centuries reflects the long-term background rate of TE invasion in *D. melanogaster*. A continuous high rate of TE invasions could result in either a gradual expansion of genome size over time or a dynamic equilibrium in which TEs continuously invade, become silenced, and get eliminated from genomes, e.g. due to a deletion bias (Petrov et al. 1996). Alternatively, the invasion rate may have increased in the last two centuries. What could cause such an elevated rate of HT during the last centuries? We think that the most plausible explanation is the recent range expansion of *D. melanogaster*, possibly mediated by anthropogenic activity. About 200 years ago, *D. melanogaster* spread from Europe and Africa to the Americas and Australia (Sturtevant 1921; Bock and Parsons 1981; Keller 2007). This range expansion brought *D. melanogaster* into contact with novel species that could act as donors of novel TEs. For example, the spread of *D. melanogaster* in the habitat of species of the *willistoni* group allowed the HT of the *P*-element, *Spoink*, and *McLE*. Range expansions will also result in an increase in population size and bring species into contact with novel vectors that could mediate the HT of TEs, thus increasing the chances for such events among species. Hence, the recent range expansion might also explain the high rate of HT between *D. melanogaster* and *D. simulans*, even though these species previously shared a common distribution for a long time (i.e. >200 years ago).

Another effect of anthropogenic activity could have been revealed by the spatiotemporal spread of *Transib1*. Strikingly, *Transib1* invaded almost all populations from Europe and North America in a mere 2 years (Fig. 3). If we assume that *D. melanogaster* has about 15 generations per year (Pool 2015 ), then *Transib1* penetrated the *D. melanogaster* populations in 30 generations. Studies that monitored TE invasions in experimental populations of different *Drosophila* species revealed that it takes about 20 generations for TEs to spread in a population (i.e. to amplify from a few copies per individual to stable high copy numbers Kofler et al. 2018, 2022; Selvaraju et al. 2024). Although the spread in geographically distributed natural populations is likely much slower than in the panmictic experimental populations, it is plausible that a TE may reach high copy number in natural populations within 30 generations. It is likely that human activity facilitated the spread of *Transib1* across different populations, for example by transporting flies as stowaways with cargo. The fact that the *Transib1* invasion commenced at three different geographically distant epicenters in 2014 is in favor of human activity (Fig. 3). TEs would likely spread slowly in the absence or with reduced human activity, as suggested by the *P*-element invasion. This invasion occurred during a period when air traffic, for example, was less intense than now and took several decades to reach all of the sampled populations (1950–1980) (Kidwell 1983; Anxolabéhère et al. 1988).

Given that many other insects species are currently expanding their distributions due to human activity and climate change (Lee et al. 2011; Tang et al. 2019; Parvizi et al. 2023), two considerations are important. First, HT is a stochastic event and will likely not occur immediately upon contact between species. As a result, TE invasions will likely lag behind range expansions. Second, range expansion of insects lag behind the range expansion of plants (many insects depend on some host plants). Given the amount of range expansions of plants, it has been estimated that many more range expansions of insects are yet to follow (Bonnamour et al. 2023). As a summed effect of these two lags, the rate of TE invasions may be increasing for many insect species in the coming decades. Thus, future research will be crucial to establish whether other insect species also show a high rate of recent TE invasions (e.g. using museomics Raxworthy and Smith 2021 ) and to continue to monitor natural populations for novel invasions with community efforts such as DrosEU (Kapun et al. 2021 ). Finally, it will be important to understand the phenotypic effects of these TE invasions, especially given the substantial impact of TEs on the evolution of genomes and phenotypes.

## Materials and Methods

### Detecting the Three New Transposon Invasions

To identify candidates of TEs that may have invaded *D. melanogaster* recently, we used our new tool, GenomeDelta (Pianezza et al. 2024). GenomeDelta is based on the idea that recent TE invasion will lead to sequences present in young strains (i.e. collected after the invasion) that are absent in old strains (collected before the invasion). To identify these sequences, GenomeDelta aligns short reads of an old strain to the genome assembly of a young strain, identifies gaps in the coverage as for example resulting from insertions of a novel TE, extracts the sequences responsible for the coverage gaps, clusters them based on homology (using a minimum bit-score of 1,000), generates a multiple sequence alignment and infers a consensus sequence (Pianezza et al. 2024). We discovered the invasions of *McLE*, *Souslik*, and *Transib1* by aligning short-read data of three old strains (SRA numbers: SRR23876563 Shpak et al. 2023, SRR11846565, SRR11846560 Schwarz et al. 2021; collected between 1810–1965) to the assemblies of 4 recently collected strains (NCBI accessions: GCA_020141595.1, GCA_020141505.1, GCA_020141485.1, GCA_020141515.1; collected between 2015–2016 Rech et al. 2022).

### Characterizing the Invasions

We generated a phylogenetic tree based on the reverse transcriptase (RT) domain of the 7 LTR retrotransposons which spread in *D. melanogaster* during the last 200 years. We selected several additional sequences from different LTR superfamily/groups as reference (Kapitonov and Jurka 2003; Quesneville et al. 2005) (v9.44). A BLASTx search against the NCBI database was conducted to identify the RT domain in the consensus sequences (Wheeler et al. 2007). The amino acid sequences of the RT domain were aligned using MUSCLE (v3.8.1551) (Edgar 2004). An XML input file for Bayesian phylogenetic analysis was generated using BEAUti2 (v2.7.5) (Bouckaert et al. 2019). Phylogenetic trees were constructed with BEAST (v2.7.5) (Bouckaert et al. 2019), and the maximum credibility tree was extracted using TreeAnnotator (v2.7.5) (Bouckaert et al. 2019).

We analyzed the population frequency of the canonical and degraded insertions for the 11 TEs that invaded recently using short-read sequencing data from pooled populations collected in Ukraine (2016; SRR8494428), the UK (2016; SRR8494432), and the USA (2014; SRR3939083) (Kapun et al. 2021). Diverged fragments were identified with RepeatMasker (Smit et al. 2013–2015) using a minimum length threshold of 300 bp and a maximum divergence of 20. The insertions frequencies were estimated using PopoolationTE2 (Kofler et al. 2016) (–min-distance −200—max-distance 300—min-count 2—max-otherte-count 2—max-structvar-count 2). We included only TE families with at least three insertions.

### Copy Number Estimates using Short-Read Data

We investigated the abundance of the 11 TEs in multiple publicly available short-read data sets. In total, we analyzed 585 strains of *D. melanogaster* (Pool et al. 2012; Grenier et al. 2015; Kapun et al. 2021; Lange et al. 2021; Schwarz et al. 2021; Rech et al. 2022; Shpak et al. 2023). For an overview of all analysed data, see supplementary file S1, Supplementary Material online. We removed pooled samples for which contamination from *D. simulans* was estimated higher than 1% (Kapun et al. 2021). To estimate the copy numbers of the TE in the samples, we used our tool DeviaTE (Weilguny and Kofler 2019). To do this, we aligned the reads to a database consisting of the consensus sequences of the 11 TEs that invaded during the last 200 years, and three single copy genes (*Antennapedia*, *Osi6*, and *Wingless*; with bwa bwasw (version 0.7.17-r1188) (Li and Durbin 2009). DeviaTE then estimates the copy number of a TE (e.g. *Souslik*) by normalizing the coverage of the TE by the coverage of the single copy genes. We also used DeviaTE to visualize the abundance and diversity of the TEs as well as to compute the frequency of SNPs in TEs.

### Copy Number Estimates in Long-read Assemblies

We investigated the abundance of the 11 TEs in long-read assemblies. The 49 analysed *D. melanogaster* strains were previously described (Pianezza et al. 2024) (data from Hoskins et al. 2015; Chakraborty et al. 2019; Wierzbicki et al. 2021; Rech et al. 2022). For an overview of all analysed assemblies, see supplementary file S2, Supplementary Material online. We identified insertions in these assemblies using RepeatMasker (open-4.0.7; -no-is -s -nolow) (Smit et al. 2013–2015).

### Identification of Diagnostic SNPs

To identify SNPs that are diagnostic for canonical insertions (insertions with high sequence identity to the consensus sequence <1.5%), we investigated the frequencies of alleles in the TE sequences. For example, if 10 insertions of a TE have the allele “A” at position 243 of the TE sequence and 5 the allele “T” frequencies of the alleles at the given site are 10/15 and 5/15 for “A” and “T,” respectively. The output of DeviaTE (Weilguny and Kofler 2019) was used to obtain the frequency of SNPs. We aimed to identify alleles of the TE sequence that are present in canonical but absent in degraded insertions (i.e. diagnostic for canonical insertions), by comparing the allele frequencies between an old (SRR8494428; collected in 1̃815) and a young sample (SRR23876563; collected in 2016). We considered a SNP to be diagnostic for canonical insertions if the minor allele frequency was >0.1 in the young and >0.95 in the old sample. With this approach, we identified multiple diagnostic SNPs for most of the 11 TEs that invaded *D. melanogaster* during the last century. Finally, we averaged the frequencies of all diagnostic SNPs for a given TE family. No reads aligned to the *P*-element and *Opus* in the old samples, preventing the calculation of allele frequencies in these samples. For these two TEs, we used frequencies of 1.0 or 0.0 to indicate the presence and absence of reads aligning to the TE, respectively. We used the diagnostic SNPs (and the copy number of the TE) to trace the invasions in the short-read data.

### Origin of HT

To identify the origin of the HT that triggered the 11 invasions, we obtained long-read assemblies of 266 drosophilids (Kim et al. 2021, 2023) and of 1225 arthropod reference genomes from NCBI. The arthropod genomes were found by filtering for “arthropoda,” “chromosome level” and “reference” at the NCBI database. The list of all analysed drosophilid and arthropod species, including the source, can be found in supplementary files S4 and S3, Supplementary Material online. We used RepeatMasker (Smit et al. 2013–2015) (open-4.0.7; -no-is -s -nolow) to identify sequences with similarity to the 11 TEs that invaded *D. melanogaster* recently in these genomes. Using a Python script, we identified the best hit for each TE in each assembly (i.e. the highest alignment score) and then estimated the similarity between this best hit and the TE using the equation $s={\mathrm{rms}}_{\mathrm{best}}/{\mathrm{rms}}_{\max}$, where ${\mathrm{rms}}_{\mathrm{best}}$ is the highest RepeatMasker score (rms) in a given assembly and ${\mathrm{rms}}_{\max}$ the highest score in any of the analysed assemblies. A s=0 indicates no similarity to the consensus sequence of the TE, whereas s=1 represent the highest possible similarity.

To generate a phylogenetic trees for the TEs, we extracted the sequences of full-length insertions (>80% of the length) from species having at least one full-length insertion using bedtools (Quinlan and Hall 2010)(v2.30.0). For each TE, a multiple sequence alignment of the insertions was generated with MUSCLE (v3.8.1551) (Edgar 2004) and a tree was generated with BEAST (v2.7.5) (Bouckaert et al. 2019).

We compared the sequence divergence of TEs among the two species likely involved in the HT with the divergence of orthologous genes. We used BUSCO (v5.5.0) (Manni et al. 2021) to obtain the sequences of orthologous genes for the species of interest, the sequence divergence was calculated for each orthologous pair using a custom Python script (genes-divergence.py), and the distributions of divergence values were plotted. In addition to orthologous genes, we calculated the sequence divergence between the two most similar insertions of the TEs in the two genomes.

## Supplementary Material

msaf143_Supplementary_Data

## Data Availability

All analyses performed in this work were documented in RMarkdown and have been made publicly available, together with the resulting figures, at GitHub https://github.com/rpianezza/Dmel-200years.

## References

[msaf143-B1] Aminetzach YT, Macpherson JM, Petrov DA. Pesticide resistance via transposition-mediated adaptive gene truncation in *Drosophila*. Science. 2005:309(5735):764–767. 10.1126/science.1112699. 16051794

[msaf143-B2] Anxolabéhère D, Kidwell MG, Periquet G. Molecular characteristics of diverse populations are consistent with the hypothesis of a recent invasion of *Drosophila melanogaster* by mobile P elements. Mol Biol Evol. 1988:5(3):252–269. 10.1093/oxfordjournals.molbev.a040491. 2838720

[msaf143-B3] Arkhipova IR . Neutral theory, transposable elements, an eukaryotic genome evolution. Mol Biol Evol. 2018:35(6):1332–1337. 10.1093/molbev/msy083. 29688526 PMC6455905

[msaf143-B4] Bargues N, Lerat E. Evolutionary history of LTR-retrotransposons among 20 Drosophila species. Mob DNA. 2017:8(1):1–15. 10.1186/s13100-017-0090-3. 28465726 PMC5408442

[msaf143-B5] Bartolomé C, Bello X, Maside X. Widespread evidence for horizontal transfer of transposable elements across *Drosophila* genomes. Genome Biol. 2009:10(2):R22. 10.1186/gb-2009-10-2-r22. 19226459 PMC2688281

[msaf143-B6] Bebber DP, Ramotowski MA, Gurr SJ. Crop pests and pathogens move polewards in a warming world. Nat Clim Chang. 2013:3(11):985–988. 10.1038/nclimate1990.

[msaf143-B7] Bock I, Parsons P. Species of Australia and New Zealand. In: Ashburner M, Carson L, Thompson JJ, editors. The genetics and biology of *Drosophila*. Vol. 3a. Oxford: Academic Press; 1981. p. 349–393.

[msaf143-B8] Bonnamour A, Blake RE, Liebhold AM, Nahrung HF, Roques A, Turner RM, Yamanaka T, Bertelsmeier C. Historical plant introductions predict current insect invasions. Proc Natl Acad Sci U S A. 2023:120(24):e2221826120. 10.1073/pnas.2221826120. 37276425 PMC10268304

[msaf143-B9] Bonnivard E, Bazin C, Denis B, Higuet D. A scenario for the hobo transposable element invasion, deduced from the structure of natural populations of *Drosophila melanogaster* using tandem TPE repeats. Genet Res. 2000:75(1):13–23. 10.1017/S001667239900395X. 10740917

[msaf143-B10] Bouckaert R, Vaughan TG, Barido-Sottani J, Duchêne S, Fourment M, Gavryushkina A, Heled J, Jones G, Kühnert D, Maio ND, et al BEAST 2.5: an advanced software platform for Bayesian evolutionary analysis. PLoS Comput Biol. 2019:15(4):e1006650. 10.1371/journal.pcbi.1006650. 30958812 PMC6472827

[msaf143-B11] Brennecke J, Aravin AA, Stark A, Dus M, Kellis M, Sachidanandam R, Hannon GJ. Discrete small RNA-generating loci as master regulators of transposon activity in *Drosophila*. Cell. 2007:128(6):1089–1103. 10.1016/j.cell.2007.01.043. 17346786

[msaf143-B12] Bucheton A, Vaury C, Chaboissier MC, Abad P, Pélisson A, Simonelig M. I elements and the *Drosophila* genome. Genetica. 1992:86(1-3):175–190. 10.1007/BF00133719. 1281801

[msaf143-B13] Capy P, Gibert P. *Drosophila melanogaster*, *Drosophila simulans*: so similar yet so different. Genetica. 2004:120(1–3):5–15. 10.1023/B:GENE.0000017626.41548.97. 15088643

[msaf143-B14] Chakraborty M, Chang C, Khost D, Vedanayagam JAJ, Liao Y, Montooth K, Meiklejohn C, Larracuente A, Emerson J. Evolution of genome structure in the *Drosophila simulans* species complex. Genome Res. 2021:31(3):380–396. 10.1101/gr.263442.120. 33563718 PMC7919458

[msaf143-B15] Chakraborty M, Emerson JJ, Macdonald SJ, Long AD. Structural variants exhibit widespread allelic heterogeneity and shape variation in complex traits. Nat Commun. 2019:10(1):4872. 10.1038/s41467-019-12884-1. 31653862 PMC6814777

[msaf143-B16] Daniels SB, Peterson KR, Strausbaugh LD, Kidwell MG, Chovnick A. Evidence for horizontal transmission of the P transposable element between *Drosophila* species. Genetics. 1990:124(2):339–355. 10.1093/genetics/124.2.339. 2155157 PMC1203926

[msaf143-B17] Dobzhansky T, Epling C. Contributions to the genetics, taxonomy, and ecology of Drosophila pseudoobscura and its relatives. Washington (DC): Carnegie Institution of Washington; 1944. p. 554.

[msaf143-B18] Edgar RC . Muscle: multiple sequence alignment with high accuracy and high throughput. Nucleic Acids Res. 2004:32(5):1792–1797. 10.1093/nar/gkh340. 15034147 PMC390337

[msaf143-B19] Ellison CE, Cao W. Nanopore sequencing and Hi-C scaffolding provide insight into the evolutionary dynamics of transposable elements and piRNA production in wild strains of *Drosophila melanogaster*. Nucleic Acids Res. 2020:48(1):290–303. 10.1093/nar/gkz1080. 31754714 PMC6943127

[msaf143-B20] Engels WR . The P family of transposable elements in *Drosophila*. Annu Rev Genet. 1983:17(1):315–344 10.1146/genet.1983.17.issue-1. 6320712

[msaf143-B21] Engels WR . The origin of P elements in *Drosophila melanogaster*. Bioessays. 1992:14(10):681–686. 10.1002/bies.v14:10. 1285420

[msaf143-B22] Finnegan DJ . Eukaryotic transposable elements and genome evolution. Trends Genet. 1989:5(4):103–107. 10.1016/0168-9525(89)90039-5. 2543105

[msaf143-B23] Gainetdinov I, Vega-Badillo J, Cecchini K, Bagci A, Colpan C, De D, Bailey S, Arif A, Wu P-H, MacRae IJ, et al Relaxed targeting rules help piwi proteins silence transposons. Nature. 2023:619(7969):394–402. 10.1038/s41586-023-06257-4. 37344600 PMC10338343

[msaf143-B24] Gladyshev E . Repeat-induced point mutation and other genome defense mechanisms in fungi. In: The fungal kingdome. Hoboken (NJ): Wiley Online Library; 2017. p. 687–699. 10.1128/microbiolspec.funk-0042-2017 PMC5607778 28721856

[msaf143-B25] Glukhov I, Kotnova A, Stefanov Y, Ilyin Y. The first complete mag family retrotransposons discovered in Drosophila. Dokl Biochem Biophys. 2016:466:1–4. 27025475 10.1134/S1607672916010014

[msaf143-B26] Grenier JK, Arguello JR, Moreira MC, Gottipati S, Mohammed J, Hackett SR, Boughton R, Greenberg AJ, Clark AG. Global diversity lines–a five-continent reference panel of sequenced *Drosophila melanogaster* strains. G3 (Bethesda). 2015:5(4):593–603. 10.1534/g3.114.015883. 25673134 PMC4390575

[msaf143-B27] Gutierrez AP, Ponti L. Analysis of invasive insects: links to climate change. In: Invasive species and global climate change. Wallingford: CABI GB; 2022. p. 50–73.

[msaf143-B28] Hoskins RA, Carlson JW, Wan KH, Park S, Mendez I, Galle SE, Booth BW, Pfeiffer BD, George RA, Svirskas R, et al The release 6 reference sequence of the *Drosophila melanogaster* genome. Genome Res. 2015:25(3):445–458. 10.1101/gr.185579.114. 25589440 PMC4352887

[msaf143-B29] Hubley R, Finn RD, Clements J, Eddy SR, Jones TA, Bao W, Smit AF, Wheeler TJ. The Dfam database of repetitive DNA families. Nucleic Acids Res. 2016:44(D1):D81–D89. 10.1093/nar/gkv1272. 26612867 PMC4702899

[msaf143-B30] Jordan IK, Matyunina LV, McDonald JF. Evidence for the recent horizontal transfer of long terminal repeat retrotransposon. Proc Natl Acad Sci U S A. 1999:96(22):12621–12625. 10.1073/pnas.96.22.12621. 10535972 PMC23018

[msaf143-B31] Kapitonov VV, Jurka J. Molecular paleontology of transposable elements in the *Drosophila melanogaster* genome. Proc Natl Acad Sci U S A. 2003:100(11):6569–6574ISSN 0027–8424. 10.1073/pnas.0732024100. 12743378 PMC164487

[msaf143-B32] Kapun M, Nunez JC, Bogaerts-Márquez M, Murga-Moreno J, Paris M, Outten J, Coronado-Zamora M, Tern C, Rota-Stabelli O, Guerreiro MPG, et al Drosophila evolution over space and time (DEST): a new population genomics resource. Mol Biol Evol. 2021:38(12):5782–5805. 10.1093/molbev/msab259. 34469576 PMC8662648

[msaf143-B33] Keller A . *Drosophila melanogaster*’s history as a human commensal. Curr Biol. 2007:17(3):R77–R81. 10.1016/j.cub.2006.12.031. 17276902

[msaf143-B34] Kidwell MG . Evolution of hybrid dysgenesis determinants in *Drosophila melanogaster*. Proc Natl Acad Sci U S A. 1983:80(6):1655–1659. 10.1073/pnas.80.6.1655. 6300863 PMC393661

[msaf143-B35] Kim BY, Gellert HR, Church SH, Suvorov A, Anderson SS, Barmina O, Beskid SG, Comeault AA, Nicole Crown K, Diamond SE, et al Single-fly assemblies fill major phylogenomic gaps across the drosophilidae tree of life. BioRxiv, preprint: not peer reviewed. 10.1371/journal.pbio.3002697 PMC11257246 39024225

[msaf143-B36] Kim BY, Wang JR, Miller DE, Barmina O, Delaney E, Thompson A, Comeault AA, Peede D, D’Agostino ER, Pelaez J, et al Highly contiguous assemblies of 101 drosophilid genomes. Elife. 2021:10:e66405. 10.7554/eLife.66405. 34279216 PMC8337076

[msaf143-B37] Kofler R, Gómez-Sánchez D, Schlötterer C. PoPoolationTE2: comparative population genomics of transposable elements using Pool-Seq. Mol Biol Evol. 2016:33(10):2759–2764. 10.1093/molbev/msw137. 27486221 PMC5026257

[msaf143-B38] Kofler R, Nolte V, Schlötterer C. The transposition rate has little influence on the plateauing level of the P-element. Mol Biol Evol. 2022:39(7):msac141. 10.1093/molbev/msac141. 35731857 PMC9254008

[msaf143-B39] Kofler R, Senti K-A, Nolte V, Tobler R, Schlötterer C. Molecular dissection of a natural transposable element invasion. Genome Res. 2018:28(6):824–835. 10.1101/gr.228627.117. 29712752 PMC5991514

[msaf143-B40] Lack JB, Cardeno CM, Crepeau MW, Taylor W, Corbett-Detig RB, Stevens KA, Langley CH, Pool JE. The Drosophila genome nexus: a population genomic resource of 623 *Drosophila melanogaster* genomes, including 197 from a single ancestral range population. Genetics. 2015:199(4):1229–1241. 10.1534/genetics.115.174664. 25631317 PMC4391556

[msaf143-B41] Lack JB, Lange JD, Tang AD, Corbett-Detig RB, Pool JE. A thousand fly genomes: an expanded Drosophila genome nexus. Mol Biol Evol. 2016:33(12):3308–3313. 10.1093/molbev/msw195. 27687565 PMC5100052

[msaf143-B42] Lange JD, Bastide H, Lack JB, Pool JE. A population genomic assessment of three decades of evolution in a natural *Drosophila* population. Mol Biol Evol. 2021:39(2):msab368. 10.1093/molbev/msab368. PMC8826484 34971382

[msaf143-B43] Lee JC, Bruck DJ, Dreves AJ, Ioriatti C, Vogt H, Baufeld P. In focus: spotted wing Drosophila, *Drosophila suzukii*, across perspectives. Pest Manag Sci. 2011:67(11):1349–1351. 10.1002/ps.v67.11. 21990168

[msaf143-B44] Le Thomas A, Rogers AK, Webster A, Marinov GK, Liao SE, Perkins EM, Hur JK, Aravin AA, Tóth KF. Piwi induces piRNA-guided transcriptional silencing and establishment of a repressive chromatin state. Genes Dev. 2013:27(4):390–399. 10.1101/gad.209841.112. 23392610 PMC3589556

[msaf143-B45] Li H, Durbin R. Fast and accurate short read alignment with Burrows–Wheeler transform. Bioinformatics. 2009:25(14):1754–1760. 10.1093/bioinformatics/btp324. 19451168 PMC2705234

[msaf143-B46] Lindsley DH, Grell EH. Genetic variations of Drosophila melanogaster. Washington (DC): Carnegie Institute of Washington Publication; 1968.

[msaf143-B47] Loreto ELS, Carareto CMA, Capy P. Revisiting horizontal transfer of transposable elements in *Drosophila*. Heredity (Edinb). 2008:100(6):545–554. 10.1038/sj.hdy.6801094. 18431403

[msaf143-B48] Machado HE, Bergland AO, Taylor R, Tilk S, Behrman E, Dyer K, Fabian DK, Flatt T, González J, Karasov TL, et al Broad geographic sampling reveals the shared basis and environmental correlates of seasonal adaptation in Drosophila. Elife. 2021:10:e67577. 10.7554/eLife.67577. 34155971 PMC8248982

[msaf143-B49] Mackay TF, Richards S, Stone EA, Barbadilla A, Ayroles JF, Zhu D, Casillas S, Han Y, Magwire MM, Cridland JM, et al The *Drosophila melanogaster* genetic reference panel. Nature. 2012:482(7384):173–178. 10.1038/nature10811. 22318601 PMC3683990

[msaf143-B50] Manni M, Berkeley MR, Seppey M, Zdobnov EM. Busco: assessing genomic data quality and beyond. Curr Protoc. 2021:1(12):e323. 10.1002/cpz1.v1.12. 34936221

[msaf143-B51] Mateo L, Ullastres A, González J. A transposable element insertion confers xenobiotic resistance in *Drosophila*. PLoS Genet. 2014:10(8):e1004560. 10.1371/journal.pgen.1004560. 25122208 PMC4133159

[msaf143-B52] Modolo L, Picard F, Lerat E. A new genome-wide method to track horizontally transferred sequences: application to *Drosophila*. Genome Biol Evol. 2014:6(2):416–432. 10.1093/gbe/evu026. 24497602 PMC3942030

[msaf143-B53] Obbard DJ, Maclennan J, Kim K-W, Rambaut A, O’Grady PM, Jiggins FM. Estimating divergence dates and substitution rates in the *Drosophila* phylogeny. Mol Biol Evol. 2012:29(11):3459–3473. 10.1093/molbev/mss150. 22683811 PMC3472498

[msaf143-B54] Parvizi E, Dhami MK, Yan J, McGaughran A. Population genomic insights into invasion success in a polyphagous agricultural pest, *Halyomorpha halys*. Mol Ecol. 2023:32(1):138–151. 10.1111/mec.v32.1. 36261398 PMC10099481

[msaf143-B55] Peccoud J, Cordaux R, Gilbert C. Analyzing horizontal transfer of transposable elements on a large scale: challenges and prospects. Bioessays. 2018:40(2):1700177. 10.1002/bies.v40.2. 29283188

[msaf143-B56] Peccoud J, Loiseau V, Cordaux R, Gilbert C. Massive horizontal transfer of transposable elements in insects. Proc Natl Acad Sci U S A. 2017:114(18):4721–4726. 10.1073/pnas.1621178114. 28416702 PMC5422770

[msaf143-B57] Petrov DA, Lozovskaya ER, Hartl DL. High intrinsic rate of DNA loss in *Drosophila*. Nature. 1996:384(6607):346–349. 10.1038/384346a0. 8934517

[msaf143-B58] Pianezza R, Haider A, Kofler R. Genomedelta: detecting recent transposable element invasions without repeat library. Genome Biol. 2024:25(1):1–16. 10.1186/s13059-024-03459-5. 39696539 PMC11656972

[msaf143-B59] Pianezza R, Scarpa A, Narayanan P, Signor S, Kofler R. Spoink, a LTR retrotransposon, invaded D. melanogaster populations in the 1990s. PLoS Genet. 2024:20(3):e1011201. 10.1371/journal.pgen.1011201. 38530818 PMC10965091

[msaf143-B60] Pool JE . The mosaic ancestry of the *Drosophila* genetic reference panel and the *D. melanogaster* reference genome reveals a network of epistatic fitness interactions. Mol Biol Evol. 2015:32(12):3236–3251. 10.1093/molbev/msv194. 26354524 PMC4652625

[msaf143-B61] Pool JE, Corbett-Detig RB, Sugino RP, Stevens KA, Cardeno CM, Crepeau MW, Duchen P, Emerson J, Saelao P, Begun DJ, et al Population genomics of sub-saharan *Drosophila melanogaster*: African diversity and non-African admixture. PLoS Genet. 2012:8(12):e1003080. 10.1371/journal.pgen.1003080. 23284287 PMC3527209

[msaf143-B62] Quah FX, Almeida MV, Blumer M, Yuan CU, Fischer B, See K, Jackson B, Zatta R, Rusuwa B, Turner GF, et al Lake Malawi cichlid pangenome graph reveals extensive structural variation driven by transposable elements. Genome Res. 2025:35(5):1094–1107. 10.1101/gr.279674.124. 40210437 PMC12047535

[msaf143-B63] Quesneville H, Bergman CM, Andrieu O, Autard D, Nouaud D, Ashburner M, Anxolabéhère D. Combined evidence annotation of transposable elements in genome sequences. PLoS Comput Biol. 2005:1(2):166–175. 10.1371/journal.pcbi.0010022. 16110336 PMC1185648

[msaf143-B64] Quinlan AR, Hall IM. BEDTools: a flexible suite of utilities for comparing genomic features. Bioinformatics. 2010:26(6):841–842. 10.1093/bioinformatics/btq033. 20110278 PMC2832824

[msaf143-B65] Raxworthy CJ, Smith BT. Mining museums for historical DNA: advances and challenges in museomics. Trends Ecol Evol. 2021:36(11):1049–1060. 10.1016/j.tree.2021.07.009. 34456066

[msaf143-B66] Rech GE, Radío S, Guirao-Rico S, Aguilera L, Horvath V, Green L, Lindstadt H, Jamilloux V, Quesneville H, González J. Population-scale long-read sequencing uncovers transposable elements associated with gene expression variation and adaptive signatures in *Drosophila*. Nat Commun. 2022:13(1):1948. 10.1038/s41467-022-29518-8. 35413957 PMC9005704

[msaf143-B67] Rubin P, Loreto E, Carareto C, Valente V. The copia retrotransposon and horizontal transfer in *Drosophila willistoni*. Genet Res (Camb). 2011:93(3):175–180. 10.1017/S0016672310000625. 21450134

[msaf143-B88] Robinson J, Thorvaldsdóttir H, Winckler W, Guttman M, Lander E, Getz G, Mesirov J. Integrative genomics viewer. Nat Biotechnol. 2011:29(1):24–26. 21221095 10.1038/nbt.1754 PMC3346182

[msaf143-B68] Sánchez-Gracia A, Maside X, Charlesworth B. High rate of horizontal transfer of transposable elements in *Drosophila*. Trends Genet. 2005:21(4):200–203. 10.1016/j.tig.2005.02.001. 15797612

[msaf143-B69] Sarkies P, Selkirk ME, Jones JT, Blok V, Boothby T, Goldstein B, Hanelt B, Ardila-Garcia A, Fast NM, Schiffer PM, et al Ancient and novel small RNA pathways compensate for the loss of piRNAs in multiple independent nematode lineages. PLoS Biol. 2015:13(2):1–20. 10.1371/journal.pbio.1002061. PMC4323106 25668728

[msaf143-B70] Scarpa A, Pianezza R, Gellert HR, Haider A, Kim BY, Lai EC, Kofler R, Signor S. Double trouble: two retrotransposons triggered a cascade of invasions in Drosophila species within the last 50 years. Nat Commun. 2025:16(1):516. 10.1038/s41467-024-55779-6. 39788974 PMC11718211

[msaf143-B71] Scarpa A, Pianezza R, Wierzbicki F, Kofler R. Genomes of historical specimens reveal multiple invasions of LTR retrotransposons in *Drosophila melanogaster* populations during the 19th century. Proc Natl Acad Sci U S A. 2023:121(15):e2313866121. 10.1073/pnas.2313866121. PMC11009621 38564639

[msaf143-B72] Schaack S, Gilbert C, Feschotte C. Promiscuous DNA: horizontal transfer of transposable elements and why it matters for eukaryotic evolution. Trends Ecol Evol. 2010:25(9):537–546. 10.1016/j.tree.2010.06.001. 20591532 PMC2940939

[msaf143-B73] Schwarz F, Wierzbicki F, Senti K-A, Kofler R. Tirant stealthily invaded natural *Drosophila melanogaster* populations during the last century. Mol Biol Evol. 2021:38(4):1482–1497. 10.1093/molbev/msaa308. 33247725 PMC8042734

[msaf143-B74] Seczynska M, Lehner PJ. The sound of silence: mechanisms and implications of hush complex function. Trends Genet. 2023:39(4):251–267. 10.1016/j.tig.2022.12.005. 36754727

[msaf143-B75] Selvaraju D, Wierzbicki F, Kofler R. Experimentally evolving *Drosophila erecta* populations may fail to establish an effective piRNA-based host defense against invading P-elements. Genome Res. 2024:34(3):410–425. 10.1101/gr.278706.123. 38490738 PMC11067887

[msaf143-B76] Shpak M, Ghanavi HR, Lange JD, Pool JE, Stensmyr MC. Genomes from historical *Drosophila melanogaster* specimens illuminate adaptive and demographic changes across more than 200 years of evolution. PLoS Biol. 2023:21(10):1–31. 10.1371/journal.pbio.3002333. PMC10569592 37824452

[msaf143-B77] Smit AFA, Hubley R, Green P. RepeatMasker Open-4.0, 2013–2015. http://www.repeatmasker.org.

[msaf143-B78] Sturtevant AH . The North American species of *Drosophila*. Nature. 1921:107:1476–4687. 10.1038/107743a0.

[msaf143-B79] Tang Q, Bourguignon T, Willenmse L, De Coninck E, Evans T. Global spread of the German cockroach, *Blattella germanica*. Biol Invasions. 2019:21(3):693–707. 10.1007/s10530-018-1865-2.

[msaf143-B80] Turbelin AJ, Malamud BD, Francis RA. Mapping the global state of invasive alien species: patterns of invasion and policy responses. Glob Ecol Biogeogr. 2017:26(1):78–92. 10.1111/geb.2017.26.issue-1.

[msaf143-B81] Wallau GL, Capy P, Loreto E, Le Rouzic A, Hua-Van A. VHICA, a new method to discriminate between vertical and horizontal transposon transfer: application to the mariner family within Drosophila. Mol Biol Evol. 2016:33(4):1094–1109. 10.1093/molbev/msv341. 26685176 PMC4776708

[msaf143-B82] Wallau GL, Ortiz MF, Loreto ELS. Horizontal transposon transfer in eukarya: detection, bias, and perspectives. Genome Biol Evol. 2012:4(8):801–811. 10.1093/gbe/evs055. PMC3516303 22798449

[msaf143-B83] Wallau GL, Vieira C, Loreto É.LS. Genetic exchange in eukaryotes through horizontal transfer: connected by the mobilome. Mob DNA. 2018:9(1):1–16. 10.1186/s13100-018-0112-9. 29422954 PMC5791352

[msaf143-B84] Weilguny L, Kofler R. DeviaTE: assembly-free analysis and visualization of mobile genetic element composition. Mol Ecol Resour. 2019:19(5):1346–1354. 10.1111/men.v19.5. 31056858 PMC6791034

[msaf143-B85] Wheeler DL, Barrett T, Benson DA, Bryant SH, Canese K, Chetvernin V, Church DM, DiCuccio M, Edgar R, Federhen S, et al Database resources of the National Center for Biotechnology Information. Nucleic Acids Res. 2007:35(suppl_1):D5–D12. 10.1093/nar/gkl1031. 17170002 PMC1781113

[msaf143-B86] Wicker T, Sabot F, Hua-Van A, Bennetzen JL, Capy P, Chalhoub B, Flavell A, Leroy P, Morgante M, Panaud O, et al A unified classification system for eukaryotic transposable elements. Nat Rev Genet. 2007:8(12):973–982. 10.1038/nrg2165. 17984973

[msaf143-B87] Wierzbicki F, Schwarz F, Cannalonga O, Kofler R. Novel quality metrics allow identifying and generating high-quality assemblies of piRNA clusters. Mol Ecol Resour. 2021:22(1):102–121. 10.1111/1755-0998.13455. 34181811

