# Supplementary material for: Spatiotemporal Tracking of Three Novel Transposable Element Invasions in Drosophila melanogaster over the Last 30 Years
Source: Mol Biol Evol. 2025 Jun 6;42(7):msaf143. doi: 10.1093/molbev/msaf143 (PMC12230796; doi:10.1093/molbev/msaf143)
Supplement: msaf143_Supplementary_Data [file msaf143_supplementary_data.zip › supplement.pdf]

Supplementary figures and tables

Supplementary figures

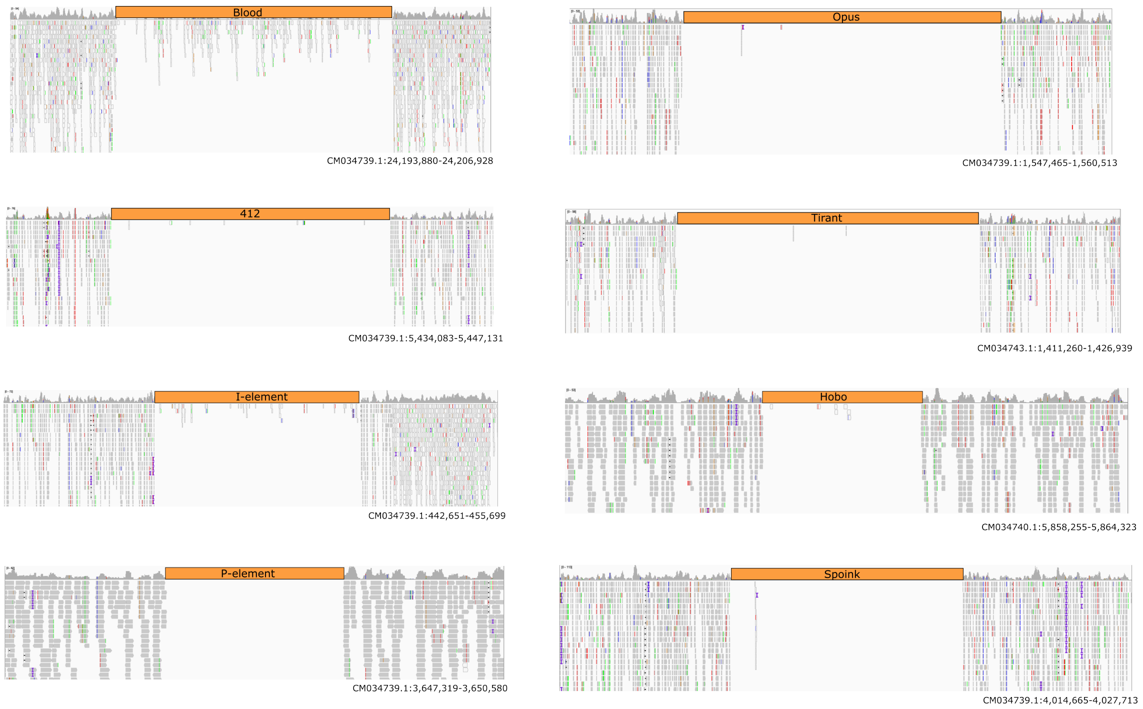

Figure 1: Example of coverage gaps caused by the 8 TE families previously shown to have invaded *D. melanogaster* populations within the last two centuries: *Blood*, *Opus*, *412*, *Tirant*, the *I*-element, *Hobo*, the *P*-element and *Spoink*. Short reads of H10 (∼1810; [Shpak et al., 2023]) were aligned to the assembly of TOM008 (2016; [Rech et al., 2022]).

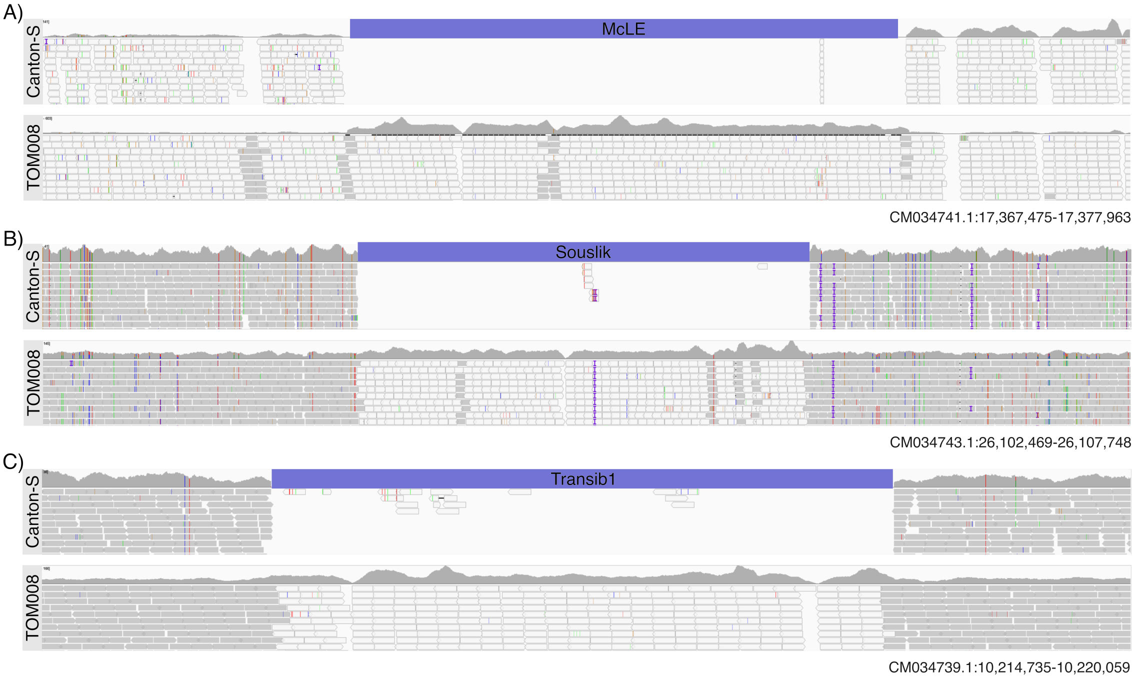

Figure 2: Example of coverage gaps caused by *McLE* (A) *Souslik* (B) and *Transib1* (C) insertions. Short reads of Canton-S (1935; [Wierzbicki et al., 2021]) were aligned to the assembly of TOM008 (2016; [Rech et al., 2022]). As control we also aligned the short-reads of TOM008 to the assembly of the same strain.

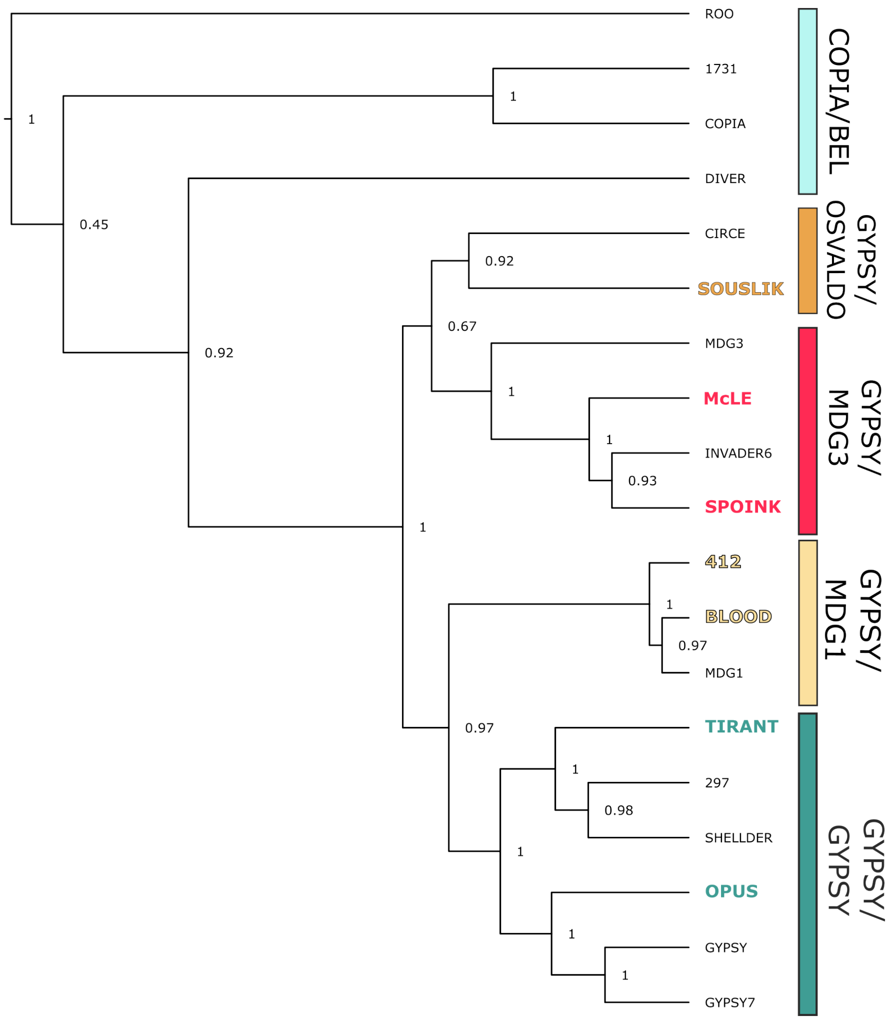

Figure 3: Phylogenetic tree of *Souslik* and *McLE* based on the reverse-transcriptase domain of *pol*. As reference we picked some families for each of the main superfamilies/groups of LTR retrotransposons and included other TEs that recently invaded *D. melanogaster* (*Tirant*, *412*, *Blood*, *Opus*) or *D. simulans* (*Shellder*) [Kapitonov and Jurka, 2003, Scarpa et al., 2023, Pianezza et al., 2023, Ding et al., 2016]. Our data suggest that *McLE* is a member of the *gypsy*/*mdg3* group and *Souslik* is a member of the *gypsy*/*osvaldo* group.

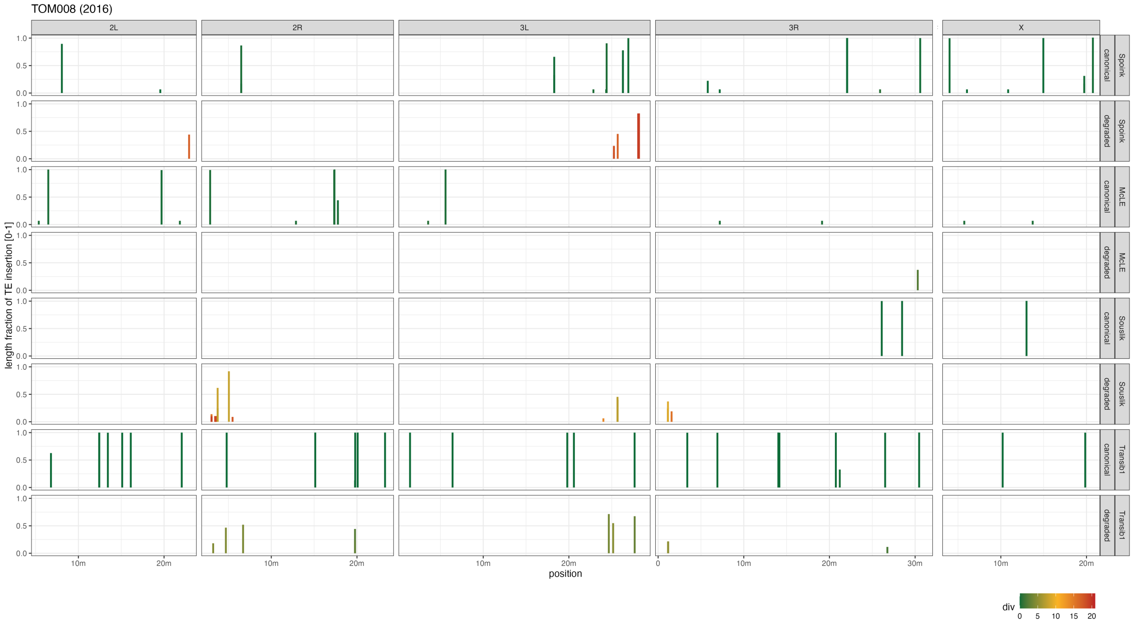

Figure 4: Overview of *Spoink*, *McLE*, *Souslik* and *Transib1* insertions in a long-read assembly of *TOM008* (identified with RepeatMasker; divergence $< 20\%$). Plots show the genomic position and the length of fragments matching with the consensus sequence of a given TE. Colors represent divergence from the consensus sequence. Based on a threshold of 1.5%, we classified the fragments into canonical ($\leq 1.5\%$) and degraded ($> 1.5\%$). The length of the TE (y-axis) is normalized to the full-length element. Note that the diverged insertions of the TEs are close to the ends of the chromosome arms (likely heterochromatic regions).

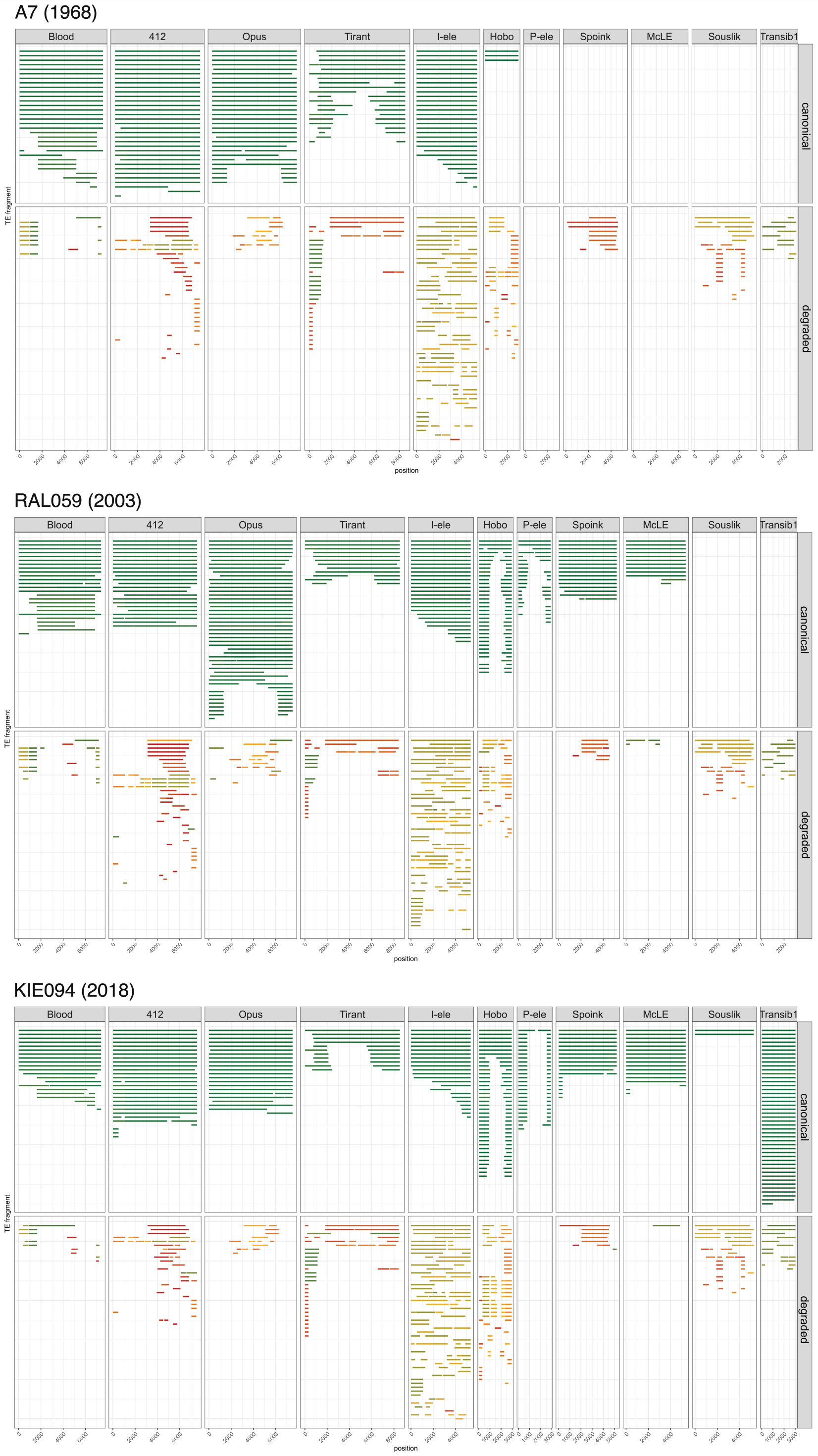

Figure 5: Regions matching with the consensus sequence of a TE in assemblies of strains collected between 1968 and 2018. Results are shown separately for canonical ($\leq$1.5% divergence) and degraded (>1.5% divergence) insertions. The number of TE families with canonical insertions increases with the sampling date of strains (the *P*-element, *Spoink*, *McLE*, *Souslik* and *Transib1*).

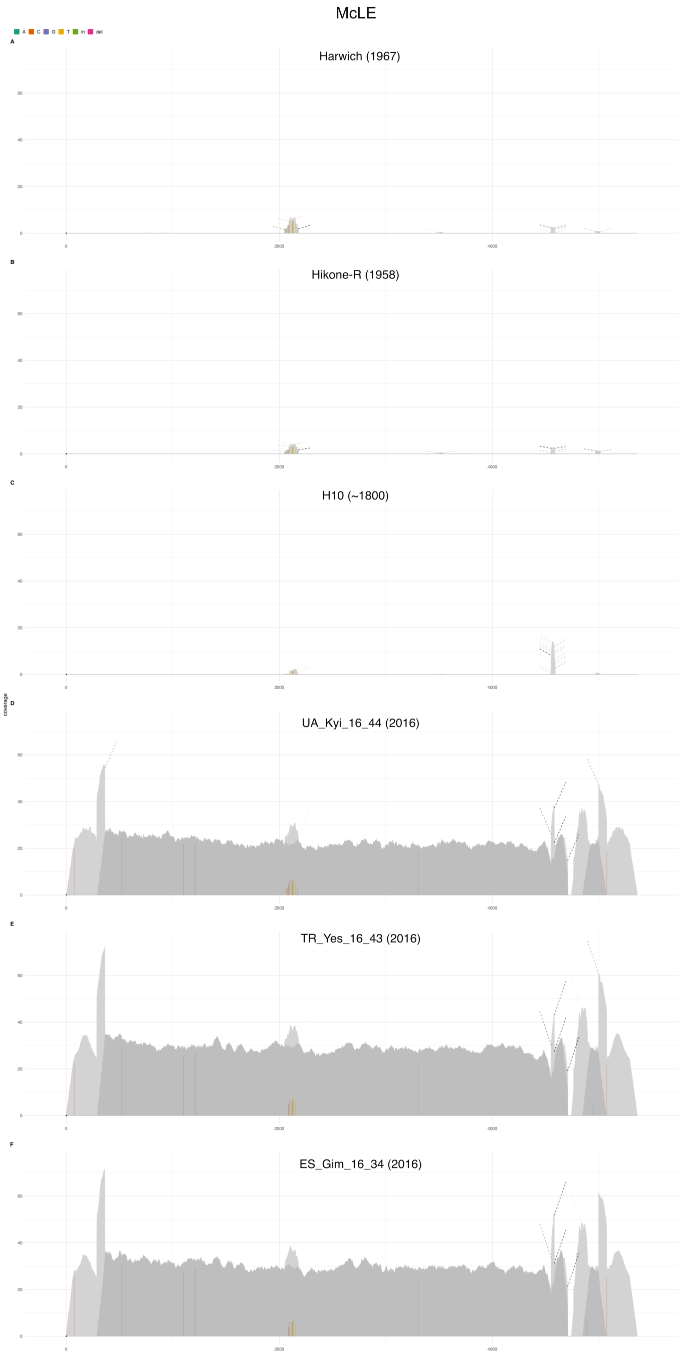

Figure 6: DeviaTE plots of *McLE* for six *D. melanogaster* strains collected during the last centuries. The short reads were aligned to the consensus sequence of TEs and the coverage was normalized to the coverage of single-copy genes. SNPs and indels are shown as colored lines. Coverage based on unambiguously and ambiguously aligned reads is shown in dark and light grey, respectively. Note that very few reads of old strains ($\leq$ 1967) align to *McLE* whereas a contiguous coverage is observed for more recently collected strains (2016).

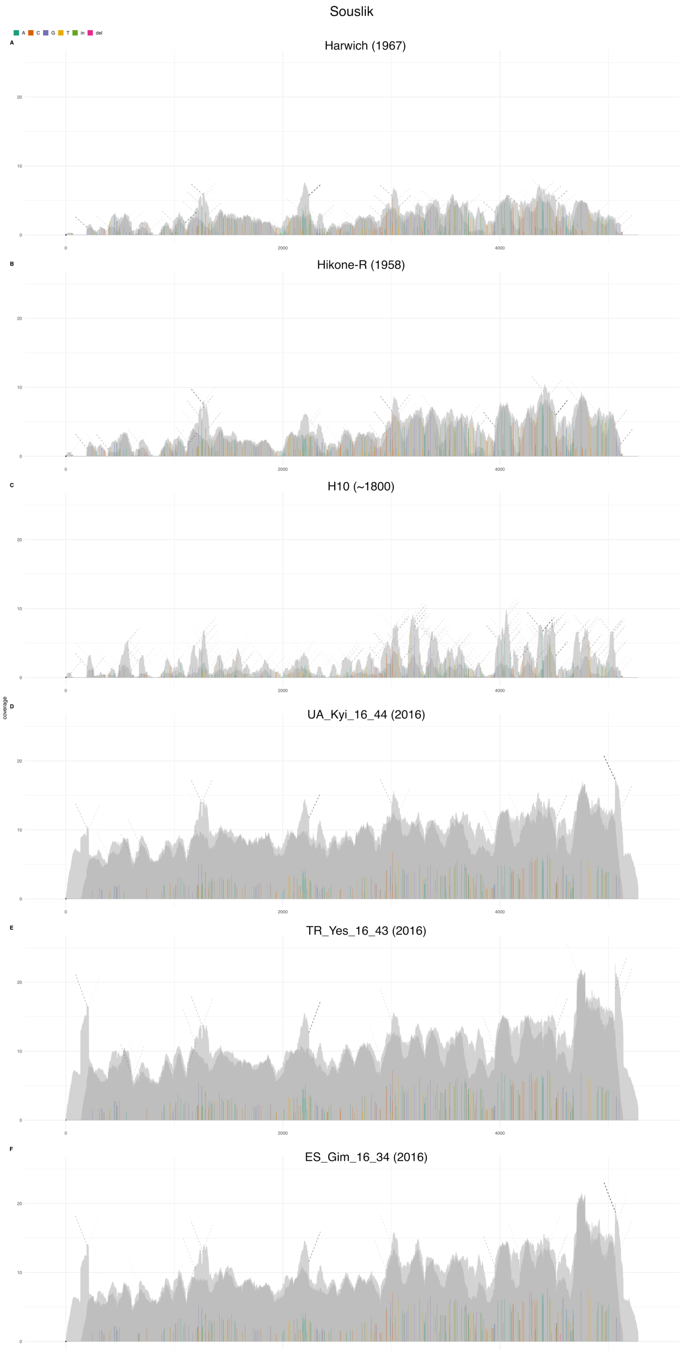

Figure 7: DeviaTE plots of *Souslik* for six *D. melanogaster* strains collected during the last centuries. The short reads were aligned to the consensus sequence of TEs and the coverage was normalized to the coverage of single-copy genes. SNPs and indels are shown as colored lines. Coverage based on unambiguously and ambiguously aligned reads is shown in dark and light grey, respectively. Note that in old strains ($\leq$ 1967) solely a few diverged reads align to *Souslik*, whereas a high contiguous coverage is observed for more recently collected strains (2016). Furthermore, the increase in coverage is largely due to reads having a high similarity to the consensus sequence of *Souslik*.

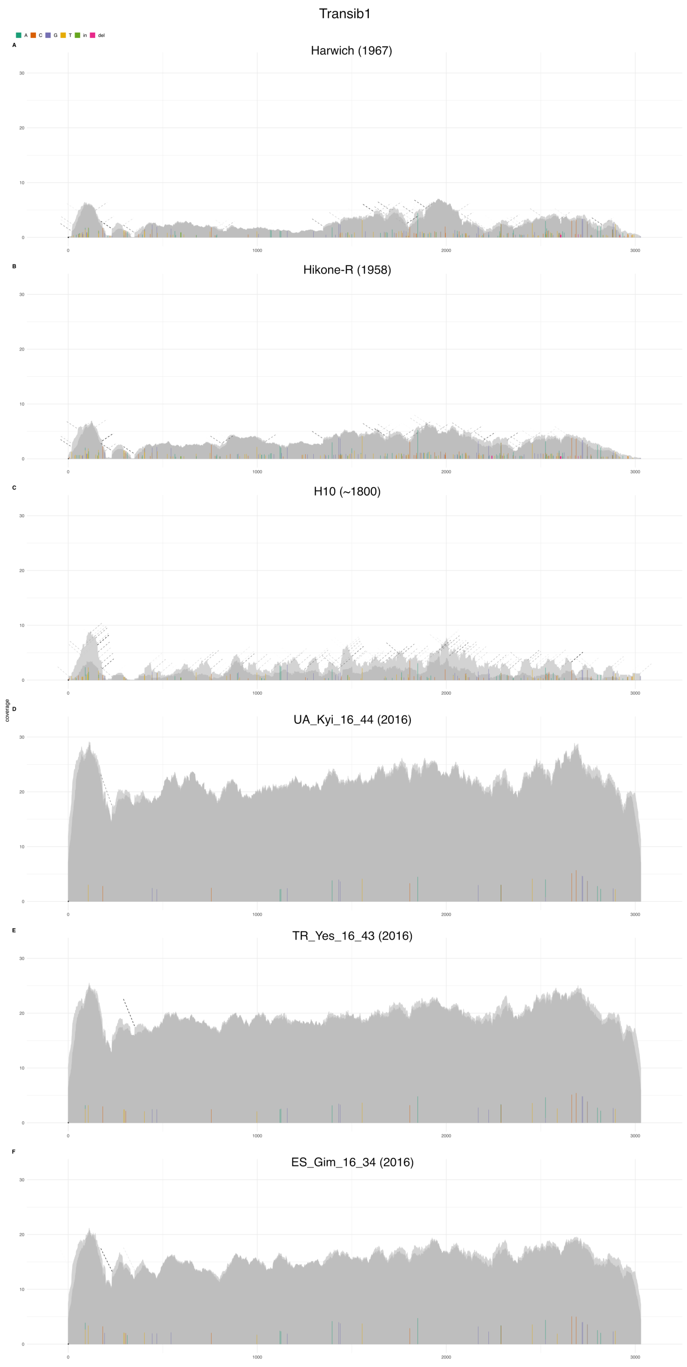

Figure 8: DeviaTE plots of *Transib1* for six *D. melanogaster* strains collected during the last centuries. The short reads were aligned to the consensus sequence of TEs and the coverage was normalized to the coverage of single-copy genes. SNPs and indels are shown as colored lines. Coverage based on unambiguously and ambiguously aligned reads is shown in dark and light grey, respectively. Note that in old strains ($\leq$ 1967) solely a few diverged reads align to *Transib1*, whereas a high contiguous coverage is observed for more recently collected strains (2016). Furthermore the increase in coverage is largely due to reads having a high similarity to the consensus sequence of *Transib1*.

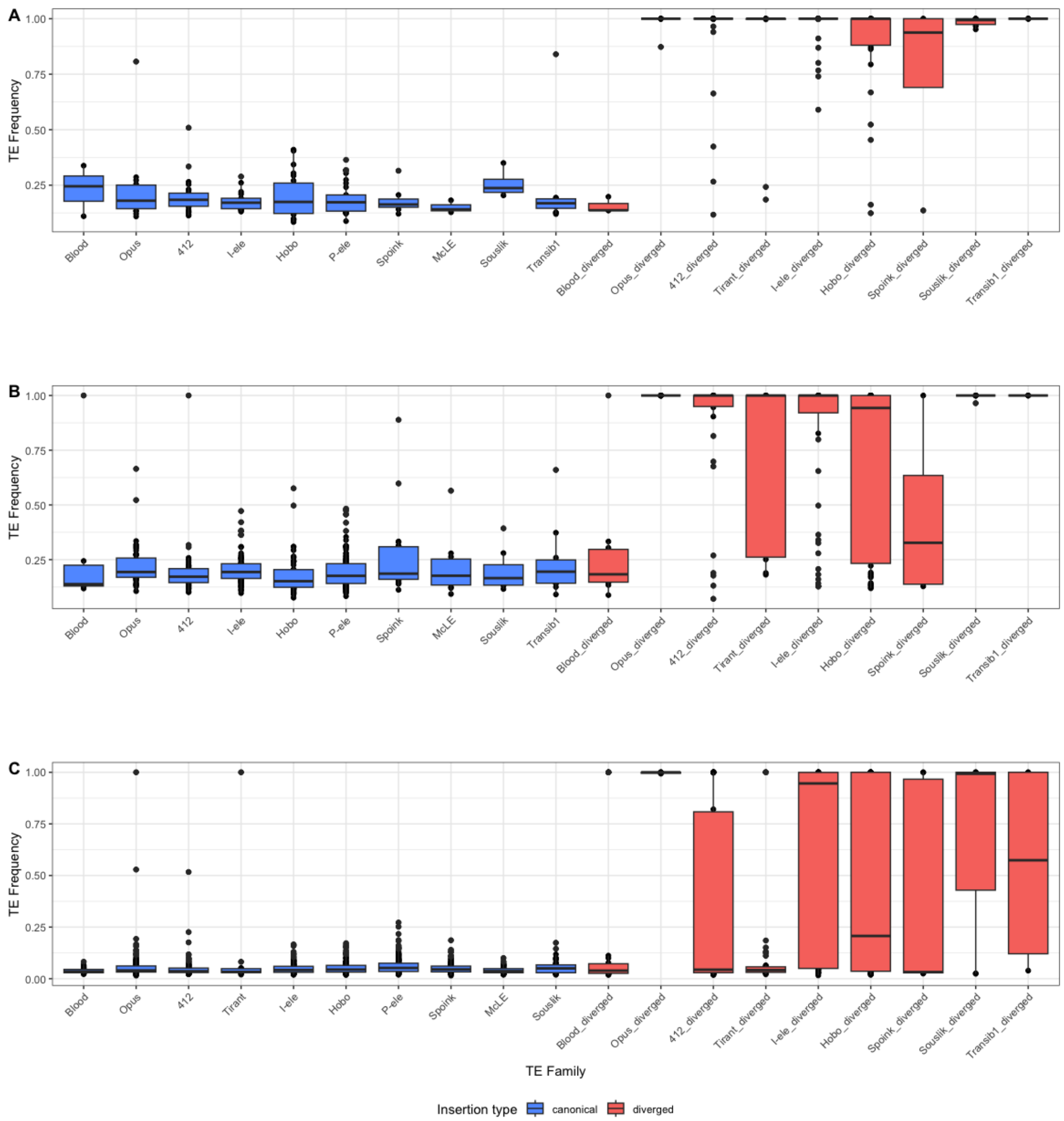

Figure 9: Population frequency of canonical (blue) and diverged (red) insertions for the 11 TEs that invaded *D. melanogaster* during the last two centuries. Population frequencies were estimated with PoPoolationTE2 [Kofler et al., 2016] based on pooled short-read data from three different populations: A) Ukraine, collected in 2016 (SRR8494428); B) United Kingdom, collected in 2016 (SRR8494432); C) United States, collected in 2014 (SRR3939083) [Kapun et al., 2021]. Canonical insertions are largely segregating at a low frequency, consistent with a recent invasion. By contrast, diverged insertions are mostly fixed, suggesting a more ancient origin (higher frequency variability may reflect their presence in heterochromatic regions, which are harder to resolve using short-read data).

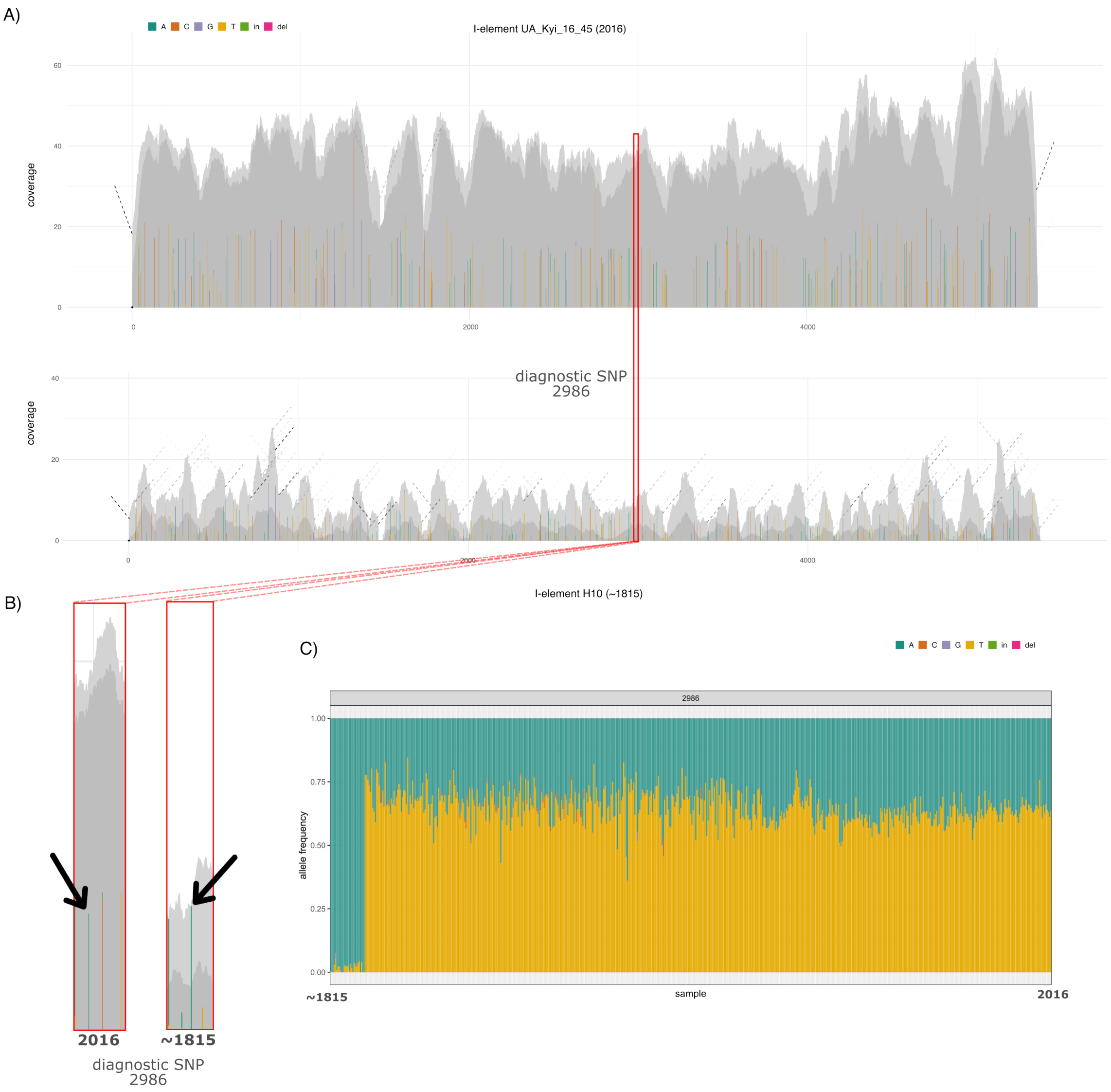

Figure 10: Diagnostic SNPs enable tracing the invasion of canonical insertions. A) DeviaTE plots for a strain collected before (∼1815) and after (2016) the invasion of the canonical *I*-element. Colored lines indicate the abundance of SNPs and indels (the reference allele is not shown). Many SNPs are segregating in the sample from 2016 (the presence of canonical and degraded insertions leads to two alleles) but are fixed in the sample from 1815 (the degraded insertions have just one allele). B) Magnification of one of these SNPs, at position 2986 of the I-element. Arrows indicate the same site. This SNP has two alleles in the sample collected in 2016, but just one in the sample collected in ∼1815. Hence, the reference allele of this SNP is diagnostic for the canonical *I*-element. C) Frequencies of the two alleles at the diagnostic SNP at position 2986 of the *I*-element in 585 *D. melanogaster* strains collected during the last two centuries. A sudden shift in the allele frequencies is observed at the time of the invasion of the canonical *I*-element. The frequency of the SNP at position 2986 can thus be used to trace the invasion of the canonical *I*-element.

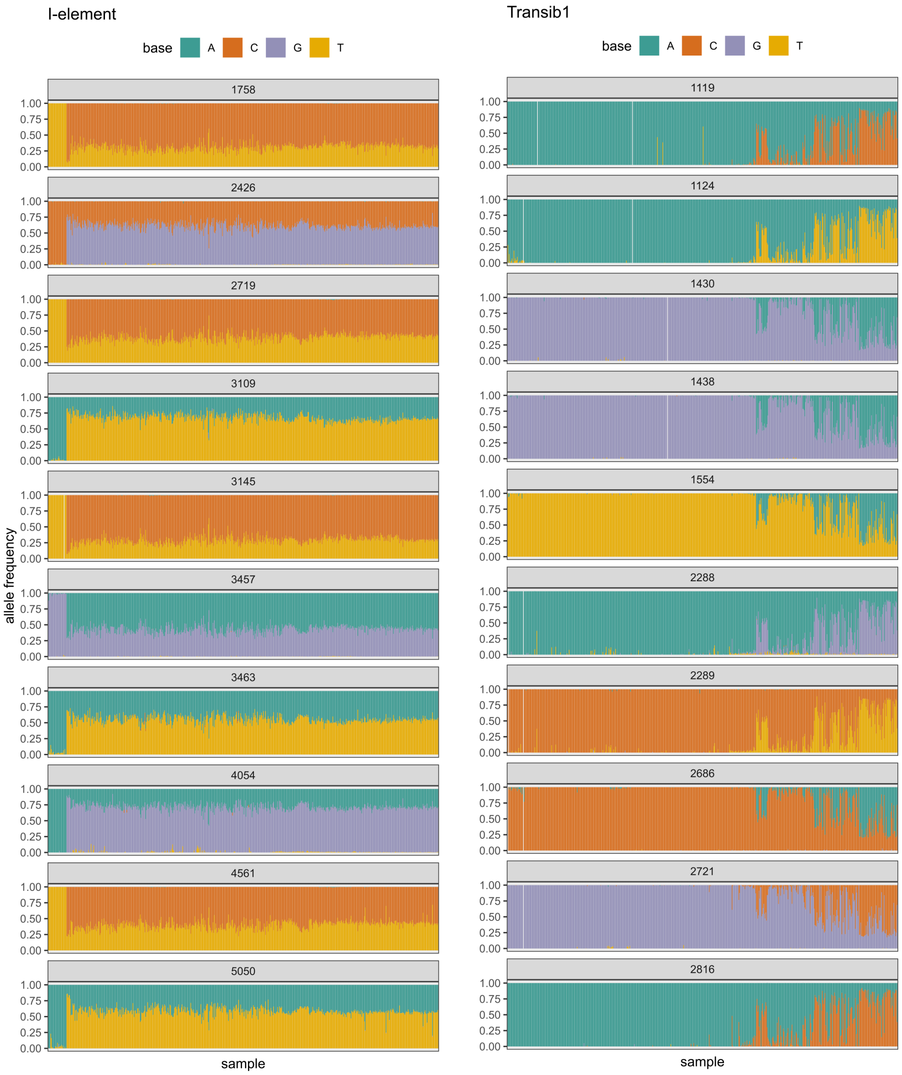

Figure 11: Diagnostic SNPs enable to consistently infer the timing of the invasion. We show the allele frequency of several SNPs that are diagnostic for canonical insertions of the *I*-element and *Transib1*. Allele frequencies are shown for 585 strains sampled during the last two centuries (ascending order by year). For each TE, ten randomly chosen diagnostic SNPs are shown (the position of the SNP in the consensus sequence is shown in the top panel). A consistent shift in the allele frequency can be observed for all diagnostic SNPs at the onset of the invasion of canonical insertions.

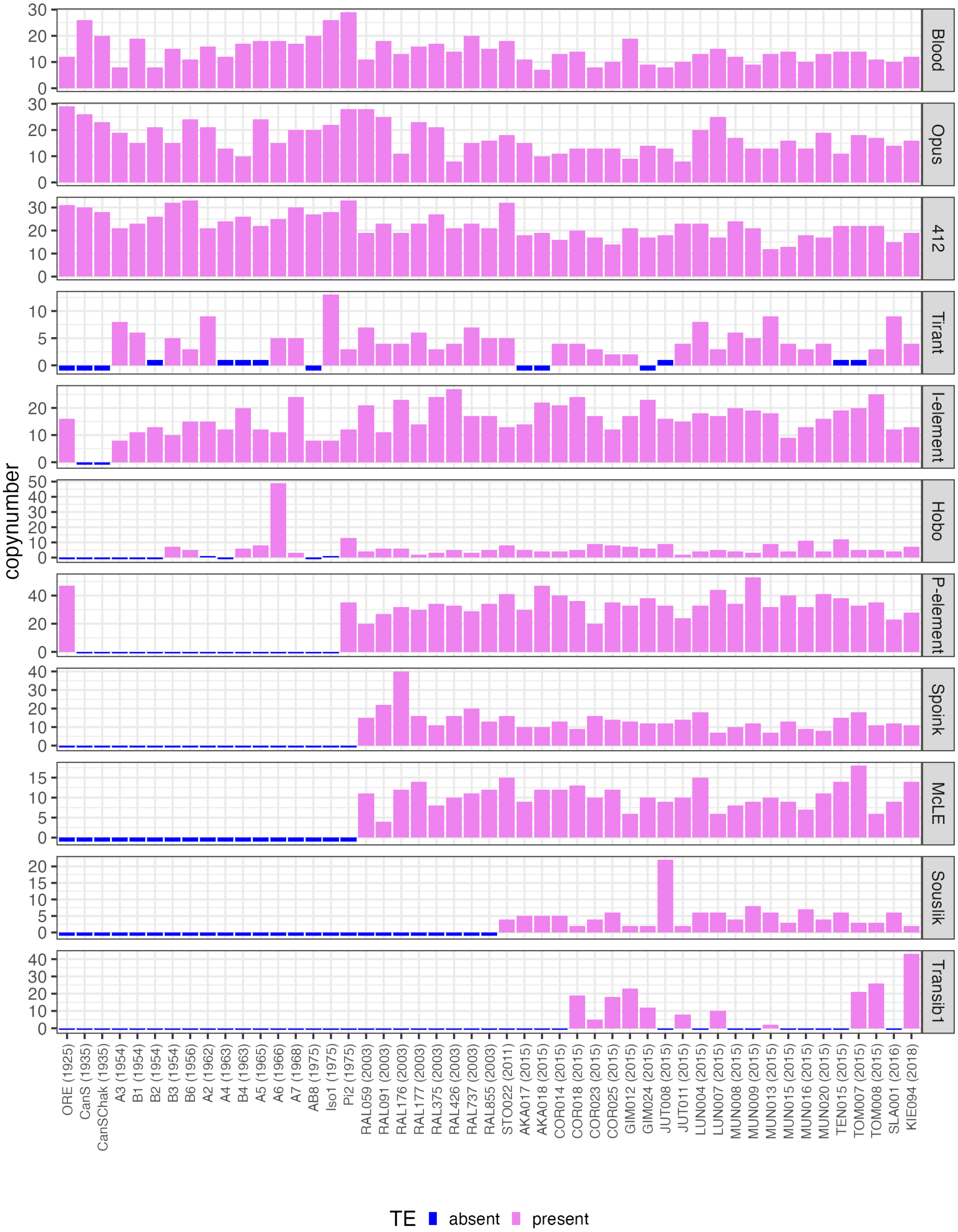

Figure 12: Abundance of canonical insertions for the 11 TEs that invaded *D. melanogaster* recently in long-read assemblies of different *D. melanogaster* strains collected between 1925 and 2018. We assumed that canonical insertions have <1.5% divergence from the consensus sequence over > 80% of the length, except for the *P*-element and *Hobo*, where we used a length threshold of 50%, to account for insertions with internal deletions [Black et al., 1987].

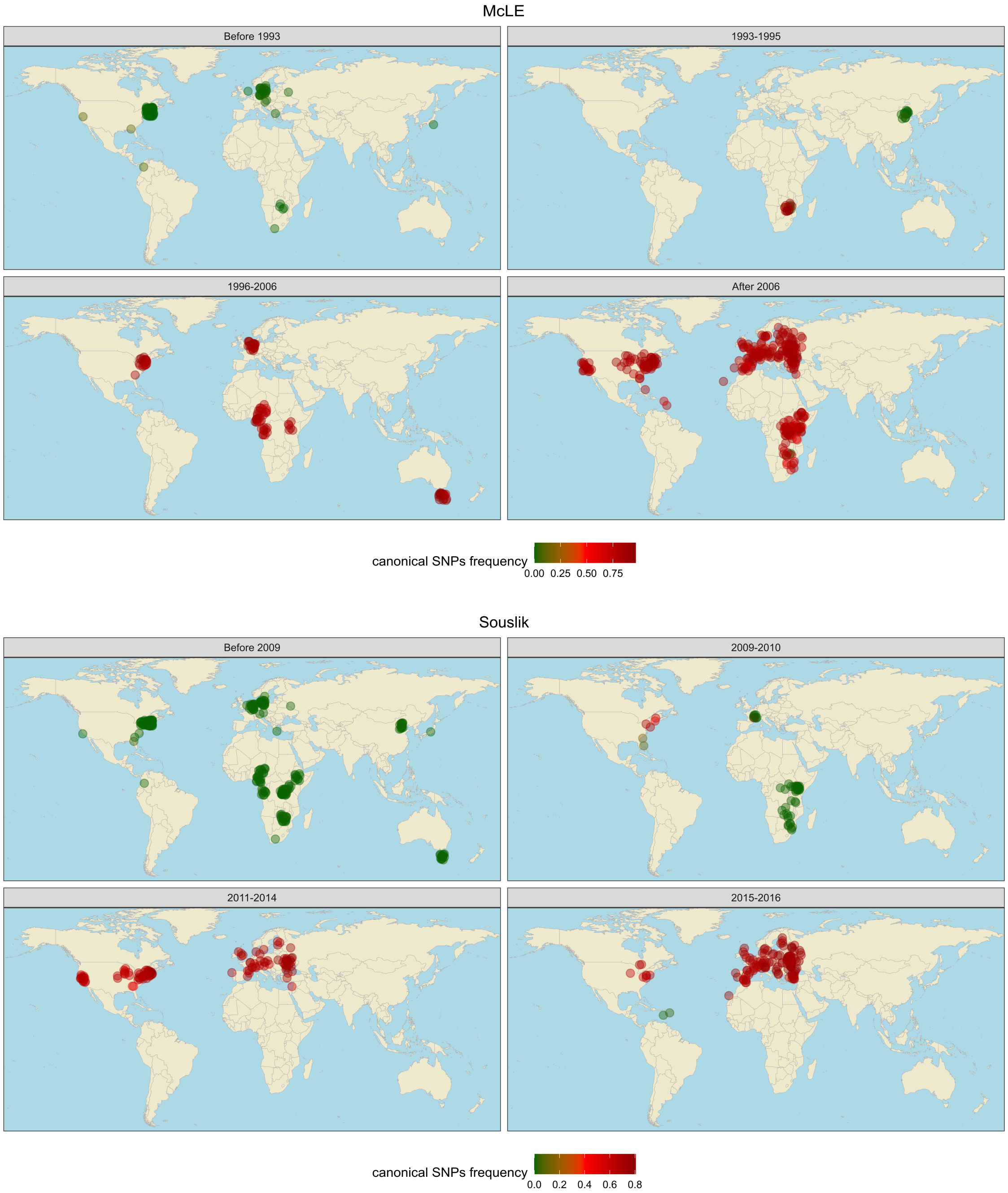

Figure 13: Geographic spread of canonical *McLE* and *Souslik* insertions in *D. melanogaster* samples (strains and pooled populations) collected at different geographic locations during the last decades. Colors of dots refers to the frequency of SNPs diagnostic for canonical insertions.

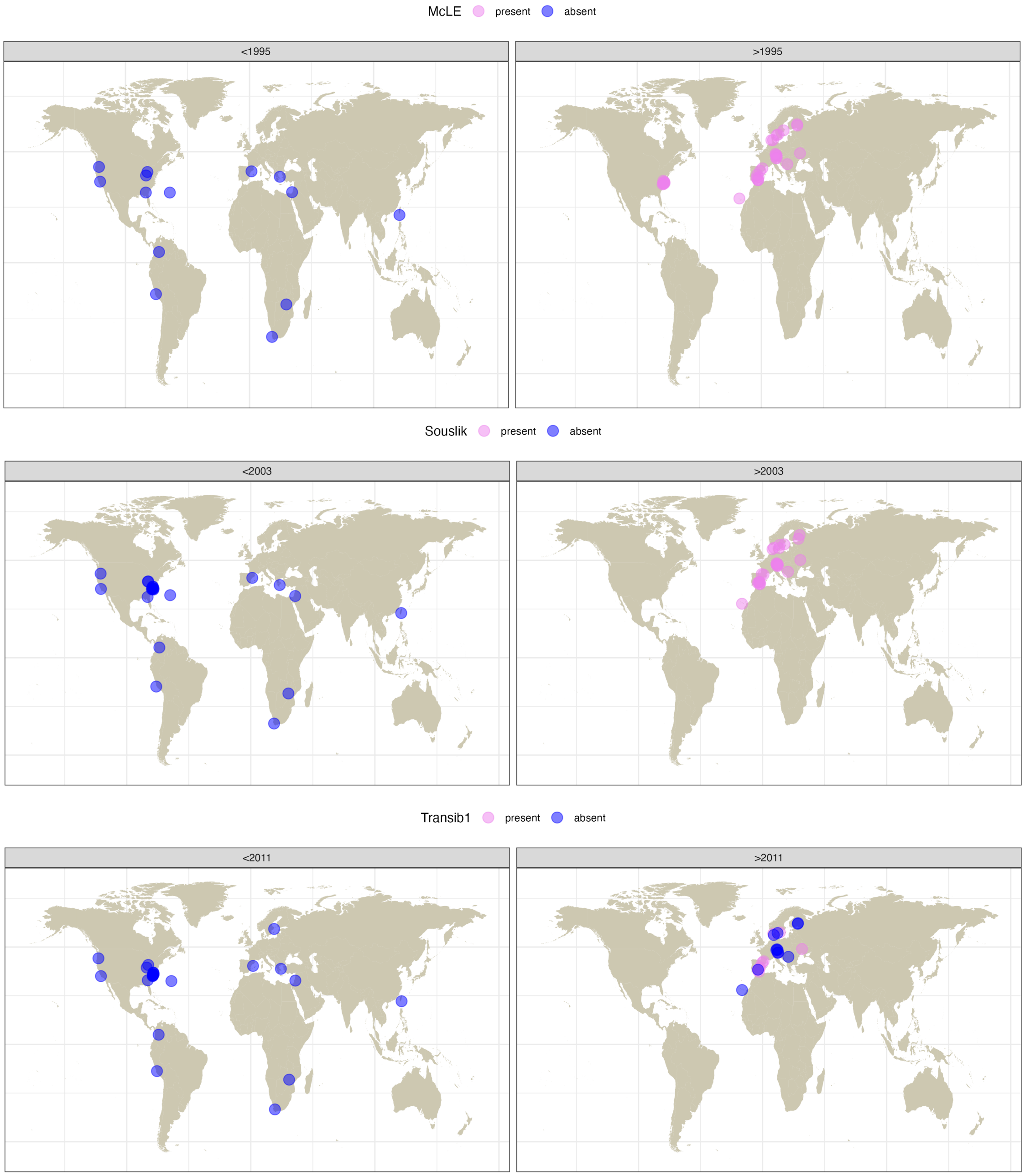

Figure 14: Geographic spread of canonical *McLE*, *Souslik* and *Transib1* insertions in long-read assemblies of *D. melanogaster* strains collected at different geographic locations during the last decades. The number of canonical insertions in the assemblies was estimated with RepeatMasker (<1.5% divergence; >80% of length). absent: number of canonical insertions is zero; present: at least one canonical insertion was found

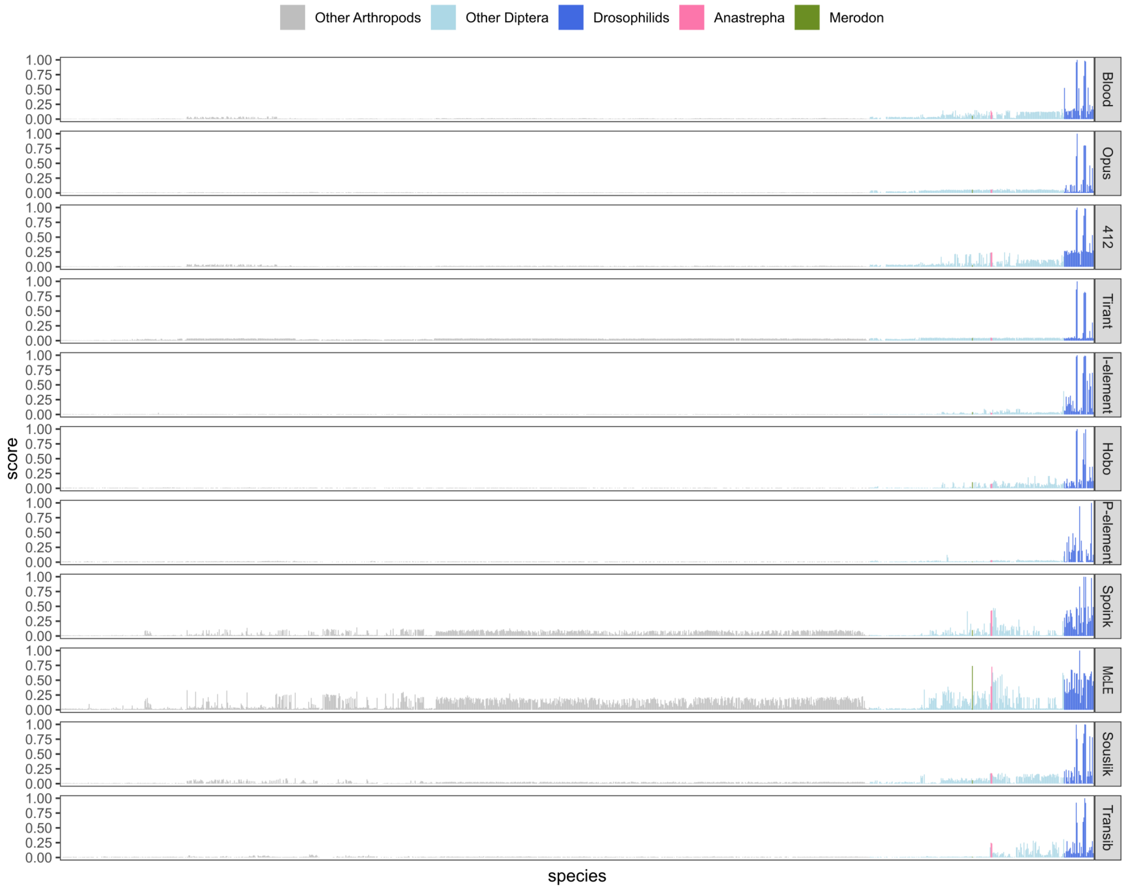

Figure 15: Similarity of the consensus sequence of the 11 TEs which spread in *D. melanogaster* recently, with TE insertions in 1226 assemblies of arthropod species. The barplots show, for each species, the similarity between the given TE and the best match in an assembly. For example, a value of 0.9 indicates that at least one insertion in an assembly has a high similarity to the given TE. For the 11 TE families, few similar sequences can be found outside of the *drosophilids*, with the exception of *McLE*, where we found similar sequences in *Anastrepha obliqua* and *Merodon equestris*

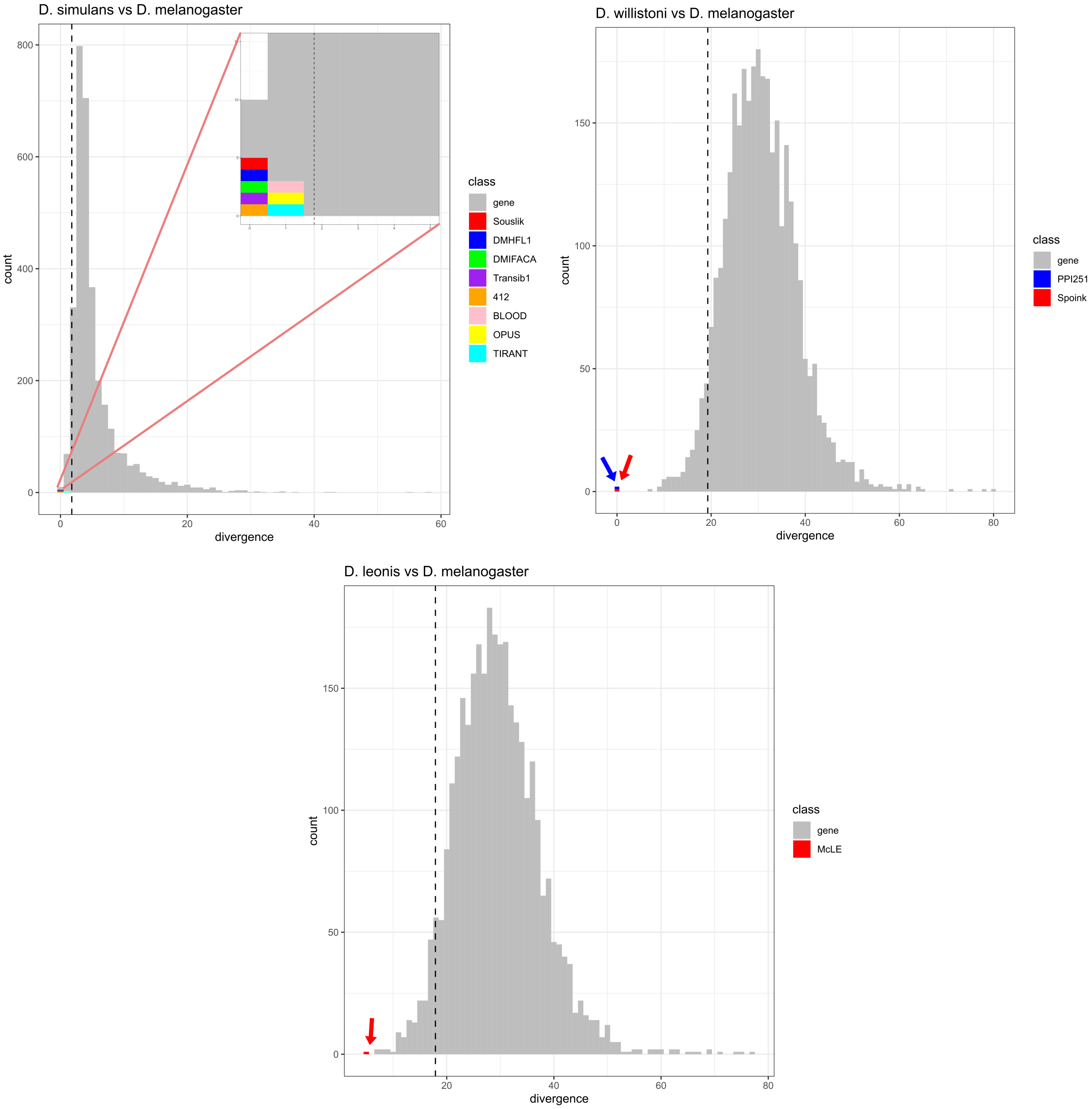

Figure 16: The sequence divergence of the TEs is lower than expected under the null-hypothesis of vertical transmission. For each of the TEs that spread in *D. melanogaster* during the last 200 years, we compared the divergence of the two most similar TE insertions between the two species that were likely involved in the HT of the TE with the divergence of the BUSCO genes. For all 11 TEs the sequence similarity is higher than for 95% (dashed line) of the BUSCO genes supporting horizontal transfer of the TEs.

Supplementary tables

Table 1: For each of the 11 TEs that spread in *D. melanogaster* during the last 200 years, we show DFAM accession ID(s) and the best match with entries in the TE library generated by Rech et al. [2022]. Percent identity (pid) and percent of the length (pl) matching with entries in Rech et al. [2022] are also reported.

TE	Dfam ID	Dfam ID (LTR)	Rech et al. 2022	pid	pl
Blood	DF000001546.3	DF000001547.3	con16_blood	98.6	78.4
412	DF000001575	DF000001576	con48_412	98.1	87.7
Opus	DF000001678.4	DF000001679.3	con25_opus	98.4	83.5
Tirant	DF000001715.4	DF000001716.3	con26_Tirant	98.5	73.8
Hobo	DF000001645.4		con43_H	95.5	100
DMIFACA	DF000001648.3		con26_I-element	97.6	100
PPI251	DF003894184.2		con33_P-element	97.8	73.9
Spoink	DF003894158.2	DF003894159.2	con41_UnFmcl001_RLX-incomp	99.0	87.4
McLE	DF003894133.2	DF003894108.2	con13_UnFmcl013_RLX-incomp	99.0	86.0
Souslik	DF003894154.2	DF003894146.2	con12_UnFmcl011_RLX-incomp	95.6	100
Transib1	DF003894177		con5_transib1	97.7	90.4

Table 2: TE content in long-read assemblies of recently collected *D. melanogaster* strains. For each of the 11 TEs that invaded *D. melanogaster* populations recently, we show the genomic proportion in base pairs. The assembly size (genome length) and the genomic fraction (in percent) occupied by these TEs are also shown.

TE	TOM008	SLA001	KIE094	COR025
412	206,031	154,672	193,658	128,098
Blood	101,936	140,926	132,474	124,489
Hobo	71,924	60,462	70,052	69,615
I-element	160,639	83,447	84,785	98,348
McLE	38,514	52,407	79,215	78,998
Opus	181,020	186,349	166,319	170,726
P-element	41,862	27,891	32,545	47,439
Souslik	15,862	41,436	10,563	35,415
Spoink	75,024	71,468	65,000	83,461
Tirant	40,188	97,229	60,994	90,723
Transib1	78,997	11,408	134,160	55,035
total	1,011,997	927,695	1,029,765	982,347
genome length	110,897,714	116,372,588	116,565,937	116,374,970
new TE fraction	0.91%	0.80%	0.88%	0.84%

References

D. M. Black, M. S. Jackson, M. G. Kidwell, and G. A. Dover. KP elements repress P-induced hybrid dysgenesis in *Drosophila melanogaster*. *The EMBO journal*, 6(13):4125–35, 1987.

Y. Ding, A. Berrocal, T. Morita, K. D. Longden, and D. L. Stern. Natural courtship song variation caused by an intronic retroelement in an ion channel gene. *Nature*, 536(7616):329–332, 2016.

V. V. Kapitonov and J. Jurka. Molecular paleontology of transposable elements in the *Drosophila melanogaster* genome. *Proceedings of the National Academy of Sciences of the United States of America*, 100(11):6569–74, 2003. ISSN 0027-8424.

M. Kapun, J. C. Nunez, M. Bogaerts-Márquez, J. Murga-Moreno, M. Paris, J. Outten, M. Coronado-Zamora, C. Tern, O. Rota-Stabelli, M. P. G. Guerreiro, et al. Drosophila evolution over space and time (dest): a new population genomics resource. *Molecular biology and evolution*, 38(12):5782–5805, 2021.

R. Kofler, D. Gómez-Sánchez, and C. Schlötterer. PoPoolationTE2: Comparative Population Genomics of Transposable Elements Using Pool-Seq. *Molecular Biology and Evolution*, 33(10):2759–2764, 2016.

R. Pianezza, A. Scarpa, P. Narayanan, S. Signor, and R. Kofler. Spoink, a ltr retrotransposon, invaded *D. melanogaster* populations in the 1990s. *bioRxiv*, 2023.

G. E. Rech, S. Radío, S. Guirao-Rico, L. Aguilera, V. Horvath, L. Green, H. Lindstadt, V. Jamilloux, H. Quesneville, and J. González. Population-scale long-read sequencing uncovers transposable elements associated with gene expression variation and adaptive signatures in *Drosophila*. *Nature Communications*, 13(1):1948, 2022.

A. Scarpa, R. Pianezza, F. Wierzbicki, and R. Kofler. Genomes of historical specimens reveal multiple invasions of ltr retrotransposons in *Drosophila melanogaster* populations during the 19th century. *PNAS*, 2023.

M. Shpak, H. R. Ghanavi, J. D. Lange, J. E. Pool, and M. C. Stensmyr. Genomes from historical drosophila melanogaster specimens illuminate adaptive and demographic changes across more than 200 years of evolution. *PLOS Biology*, 21(10):1–31, 10 2023. doi: 10.1371/journal.pbio.3002333. URL https://doi.org/10.1371/journal.pbio.3002333.

F. Wierzbicki, F. Schwarz, O. Cannalonga, and R. Kofler. Novel quality metrics allow identifying and generating high-quality assemblies of piRNA clusters. *Molecular Ecology Resources*, 2021. doi: 10.1111/1755-0998.13455.
